# Modulation of rhizosphere microbiota by *Bacillus subtilis* R31 enhances long‐term suppression of banana Fusarium wilt

**DOI:** 10.1002/imo2.70006

**Published:** 2025-03-18

**Authors:** Ming‐Wei Shao, Hao‐Jun Chen, Ai‐Qin Huang, Li Zheng, Chun‐ji Li, Di Qin, Yun‐Hao Sun, Zheng Lin, Gang Fu, Yan‐Hong Chen, Yong‐Jian Li, Zhang‐Yong Dong, Ping Cheng, Heru Pramono, Guo‐Hui Yu, Zhi‐Min Xu, Shuang Miao, Kevin D. Hyde

**Affiliations:** ^1^ College of Agriculture and Biology, Key Laboratory of Green Prevention and Control on Fruits and Vegetables in South China Ministry of Agriculture and Rural Affairs, Zhongkai University of Agriculture and Engineering Guangzhou China; ^2^ Guangxi Key Laboratory of Biology for Crop Diseases and Insect Pests/Key Laboratory of Green Prevention and Control on Fruits and Vegetables in South China Ministry of Agriculture and Rural Affairs Nannin China; ^3^ Zhuhai Modern Agriculture Development Center Zhuhai China; ^4^ Laboratory of Fisheries Microbiology, Department of Marine Science, Faculty of Fisheries and Marine Universitas Airlangga Surabaya Indonesia; ^5^ Key Laboratory for Agro‐ecological Processes in Subtropical Regions, Institute of Subtropical Agriculture Chinese Academy of Sciences Changsha China; ^6^ Center of Excellence for Fungal Research Mae Fah Luang University Chiang Rai Thailand

**Keywords:** biocontrol, *Bacillus subtilis* R31, Fusarium wilt, microbial succession, rhizosphere microbiome

## Abstract

Continuous cropping of bananas leads to Fusarium wilt, affecting crop health, yet employing biocontrol bacteria to modulate rhizosphere microbial communities may offer effective disease suppression. This study revealed the secondary succession characteristics of rhizosphere microbiota in banana fields after more than 5‐year continuous cropping and assessed the suppressive effects induced by biocontrol strain *Bacillus subtilis* R31. Through high‐throughput sequencing, we observed a convergent enrichment of core bacterial genera *Burkholderia*‐*Dyella* and *Arthrobacter*‐*Ralstonia* in naturally suppressive and R31‐treated suppressive soils, indicating broad‐spectrum disease‐suppressive soil traits induced by R31. R31 significantly enhanced associations between *Streptomyces* and the rhizosphere core community while weakening *Burkholderia*'s linkage with membrane transport and energy metabolism pathways; moreover, it strengthened positive correlations between *Rhizobium* and terpenoid and polyketide metabolism (*r* = 0.65, *p* < 0.01). Culture‐dependent assays showed that among 46 isolates from root and rhizosphere, those with high activities of indole‐3‐acetic acid, protease, cellulase, chitinase, and *β*‐1,3‐glucanase exhibited pronounced antagonistic activities against *Fusarium oxysporum*. Although R31 was undetectable in the rhizosphere in the second year, its modulation of rhizosphere function persisted, displaying a “legacy effect” consistent with the priority effects theory, whereby early colonizing beneficial communities resist subsequent pathogen invasions. R31 induced functional bacteria colonization in nascent banana root hair tissues, establishing new microbiome patterns that contributed to long‐term disease suppression. Pot experiments further indicated that endophytic bacteria within roots exhibited stronger Fusarium wilt control than rhizosphere isolates. However, single‐strain treatments frequently led to sheath rot co‐infection, suggesting that synergistic actions of multiple strains might be more effective for disease suppression. This study highlighted R31's potential to sustainably modulate the rhizosphere microbiome, enhance enzyme activity, and promote beneficial bacteria colonization, laying the groundwork for constructing efficient synthetic bacterial communities for biocontrol.

## Highlights

- *B. subtilis* R31's modulation of the rhizosphere endures even after its disappearance, enhancing pathogen resistance.
- Biocontrol bacterium R31 modulates core rhizosphere microbial genera and functional pathways related to disease suppression.
- R31 effectively controls banana Fusarium wilt by restructuring the rhizosphere microbial community, especially increasing Actinomyces abundance.
- Endophytic bacteria in roots demonstrate greater effectiveness against Fusarium wilt, emphasizing the importance of strain interactions for biocontrol.

## Graphical abstract

This study indicated that biocontrol bacterium *B. subtilis* R31 is likely to prevent and control banana Fusarium wilt through regulating the structure and function of banana rhizosphere microorganism flora (especially increasing the microbial abundance of Actinomyces), and help potential biocontrol bacteria grow into the plant roots. There is a convergent secondary succession in microbial communities between naturally suppressive soil and biocontrol R31‐treated soil.

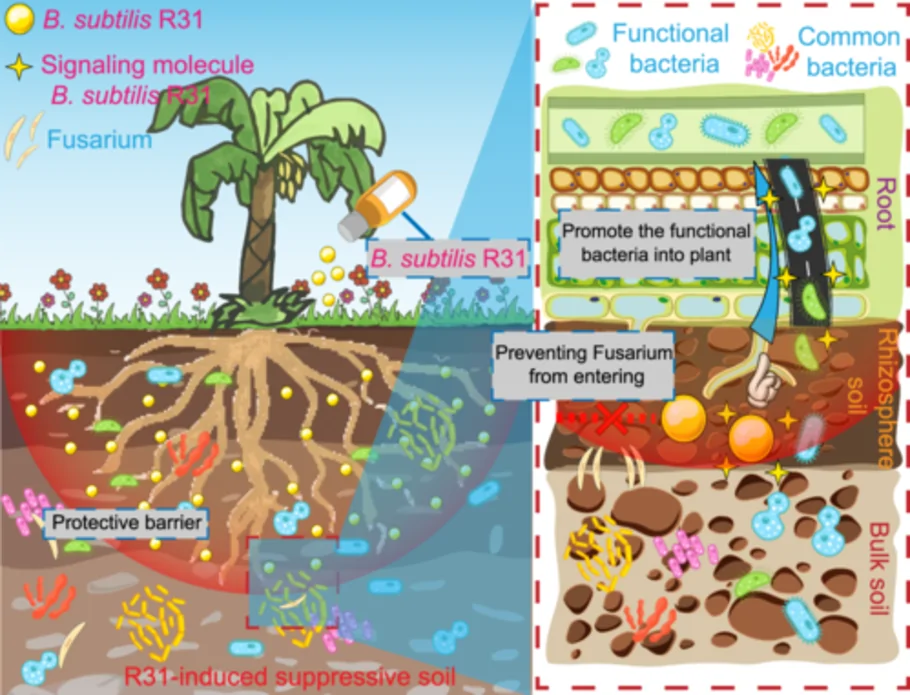

## 1 INTRODUCTION

Soil microbes benefit plant health much like probiotics or the immune system benefit human health [1, 2]. Plants can stimulate and use these microbial communities to protect themselves and resist pathogenic bacteria when they are infected [2]. Continuous planting of a single crop on the same farmland can lead to severe soil degradation due to the accumulation of detrimental exudates and residues from crop roots and pathogenic microorganisms in the soil, gradually increasing the susceptibility to disease [3, 4]. In addition, the resulting imbalances in the microbial community structure can cause decreased microbial diversity, and will reduce the ability of the soil to restrain or inhibit harmful microorganisms [5, 6]. However, in regions with plants suffering from severe disease symptoms, some areas show mild or no disease independently of crop resistance or cultivation measures [7]. In these areas, the soil has an antibacterial effect, and is known as suppressive soil. Indeed, the plant rhizosphere of suppressive soil that inhibits the pathogenic fungus *Rhizoctonia solani* is rich in beneficial Proteobacteria, Firmicutes and Actinobacteria [8]. In many cases, plants can stimulate the production of these beneficial microbial communities and thereby improve their resistance to pathogens. Microorganisms that cause disease suppression can be divided into normal and specialized types [2]. The normal disease‐suppressing soil cannot be transferred to different soils. The specific disease suppression, also known as induction type disease suppression (induced suppression), usually through certain agronomic measures regulation can be formed. Both normal and specialized types of soil disease inhibition are the manifestations of soil immunity [9, 10].

Current issues in agriculture, such as soil degradation and reduced microbial diversity, are largely due to improper fertilization and continuous cropping practices, which increase susceptibility to diseases like Fusarium wilt in banana plantations. Fusarium wilt, caused by the soil‐borne fungus *Fusarium oxysporum* f. sp. *cubense*, is a devastating disease in banana plantations worldwide [11]. It severely affects plant vascular systems, leading to wilting and eventual death of infected plants, causing significant economic losses [12]. The lack of effective chemical treatments has made biological control a critical alternative in managing this disease [13]. Plant growth‐promoting rhizobacteria can produce substances that antagonize pathogens and thereby impart host resistance, promoting plant growth and quality while enhancing and stabilizing food production and maintaining the stability of natural ecosystems [14, 15, 16]. Those microorganisms in the disease‐suppressing soil can compete with soil‐borne disease agents for nutrients, infection sites, or colonization of host plants. Biocontrol agents, such as *Bacillus* species, have been widely studied for their ability to suppress plant pathogens and promote plant health [1]. Among them, the endophytic *Bacillus subtilis* R31 has shown significant potential in controlling Fusarium wilt in banana plantations.

In banana plantations, continuous monoculture can lead to soil degradation, microbial imbalance, and increased susceptibility to diseases like Fusarium wilt. The applying of biocontrol *B. subtilis* R31, to a certain extent, can alleviate those symptoms, which form the induced suppressive soil for banana plantations. The “amplication‐selection” assembly model of the rhizosphere microbiome indicated that microorganisms are thought to be gradually selected in the soil, rhizosphere, and internal roots to form plant rhizosphere‐specific microbial communities [17]. We propose that the applying of biocontrol bacteria, as the important agronomic measures, can provide the transferability of some functional bacteria from bulk soil to the root or rhizosphere, which can form the induced suppression. This study aims to investigate how *B*. *subtilis* R31 modulates microbial communities in banana rhizosphere soil and enhances disease resistance through secondary succession. The endophytic *B. subtilis* R31 bacterium produced by Guangdong Geolong Biotechnology Co., Ltd. under the trade name “Dingwei” has been popularized and applied in major banana‐producing areas to control disease. Since 2019, the use of this bacterium in agricultural settings has yielded tremendous economic benefits through preventing Fusarium wilt of banana [12]. Over a 2‐year study, we investigated the effects of applying biocontrol R31 bacteria on the secondary succession of microorganisms in banana rhizosphere soil compared with suppressive soil. We assayed enzyme production activity, kin discrimination ability and antagonism among isolated epiphytic and endophytic bacteria in vitro and in vivo to elucidate how healthier endophytic bacterial communities in plant hosts are induced by the biocontrol bacterium *B. subtilis* R31.

## 2 RESULTS

### Banana rhizosphere microbial diversity

**FIGURE 1:**
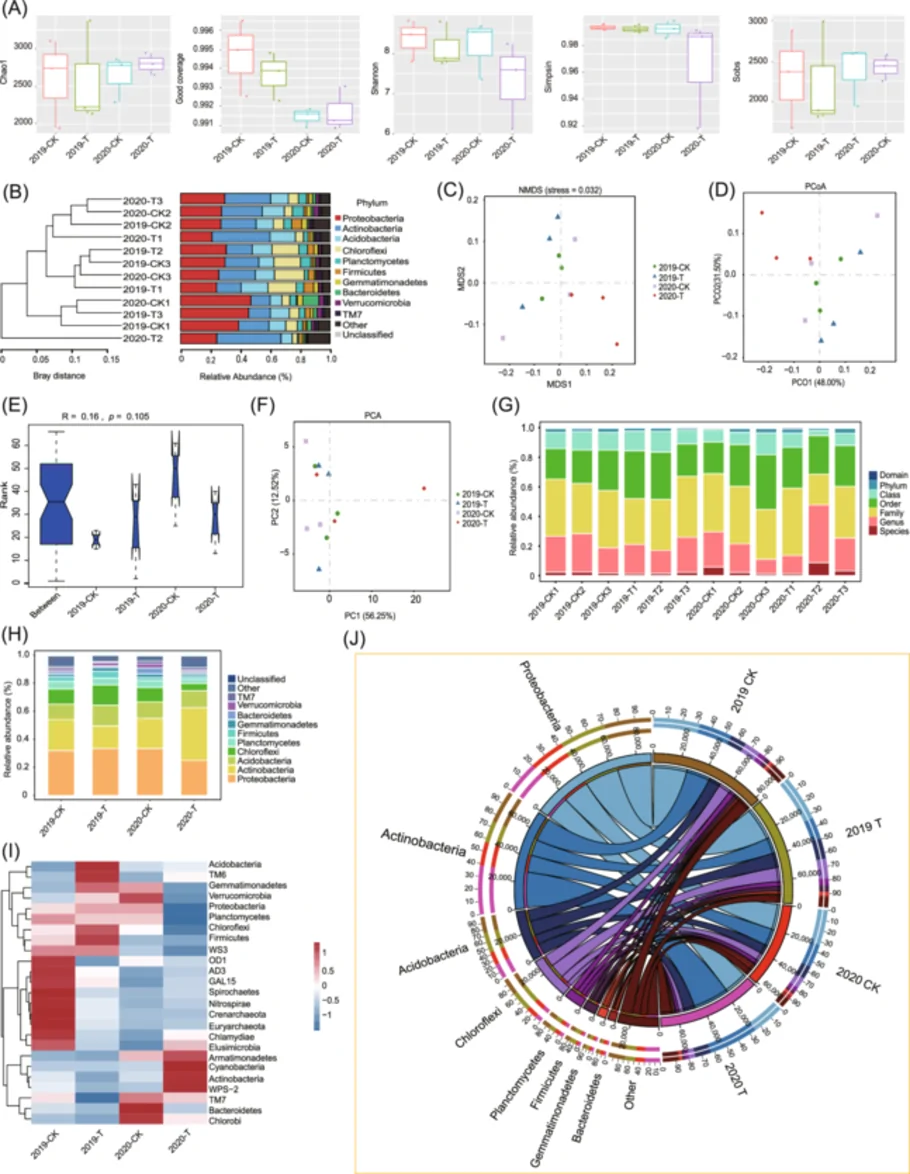
Microbiome diversity in banana rhizosphere samples. Samples of 2019‐T and 2020‐T were collected from the roots of Brazilian banana plants grown in Sanshui, Foshan and associated rhizosphere soil following a one‐time application (in 2019) of Dingwei suspension concentration. Samples of 2019‐CK and 2020‐CK were collected from naturally healthy banana roots and rhizosphere soil growing for 5 years in the absence of disease. (A) Alpha diversity measured by the Chao1, Good coverage, Shannon, Simpson, and Sobs indices. *p > 0.05, Nonparametric tests. (B) UPGMA cluster plot of Beta diversity index measured by the Bray–Curtis distance between samples. (C) Scatter plot of nonlinear models (NMDS) based on the Beta diversity index between samples. (D) Principal coordinate analysis (PCoA) plot illustrating the beta‐diversity metrics measured by the Bray–Curtis distance. (E) Analysis Of similarity (ANOSIM) to test the statistical significance between different groups. (F) Principal component analysis (PCA) illustrating the beta‐diversity metrics measured by the Bray–Curtis distance. (G) The sequence of each sample at the phylum classification level constitutes a bar graph (percentage). The abscyssus of the stacked plot shows the grouping of different samples, and the columns of different colors indicate the relative abundance of OTUs contributed by different species to the same phenotype. (H) Stacked plot of species distribution at phylum level. (I) Taxonomic heatmap of species at the taxonomic level below phylum. (J) Circos plot at the level of the grouping phylum classification. In the figure, the left semicircles represent species, and the right semicircles represent samples/groups. The width of the line from species to grouping indicates the relative abundance of species in the grouping. The outer ring of the left semicircle, the color indicates the groups, the length of the ring indicates the abundance of each species in each group, the outer ring of the right semicircle, the color indicates the species, and the length of the ring indicates the abundance of each species in each group. OTUs, operational taxonomic units.

To investigate the differences and associations in bacterial communities in suppressive soil with continuous cropping of banana for more than 5 years and soil treated with biocontrol bacteria, we performed 16S rDNA amplicon sequencing to document the changes in rhizosphere bacterial communities of *B. subtilis* R31‐treated and control samples in years 2019 and 2020. After sequencing, an average of 109,256 effective tags (ranging from 93,746 to 120,295) for each sample (Figure S1, Tables S1 and S2) were obtained. These effective tags clustered into a total of 7036 operational taxonomic units (OTUs), with an average of 2331 OTUs per sample (Figure S2 and Tables S3–S6). The *α* diversity of the microbiome based on these OTUs, including richness and Shannon and Simpson indices were estimated (Figures S3 and S4, Tables S7 and S8). There was no significant difference in *α* diversity between R31‐treated and control groups in either 2019 or 2020 (Tukey's multiple range test, *p* > 0.05; Figure 1A,B). Based on the relative abundance of OTUs, we used principal coordinates analysis (PCoA) and nonlinear models (NMDS) to analyze the source of differences in microbial community composition among samples (Figure 1C,D). The two main coordinates explained 46% of the variation. The analysis of similarity and principal component analysis showed that the distribution of the samples is not significantly different between R31‐treated and control groups or between different years (PERMANOVA, *R* = 0.022, *p* = 0.426; Figure 1E,F). Notably, there was a convergent secondary succession of the rhizosphere bacterial community during naturally suppressive soil and R31‐induced suppressive soil.

### Composition of the bacterial community induced by R31

The OTUs against the SILVA database (https://www.arb-silva.de/) were queried to obtain taxonomic classifications. Notably, there were no OTU belonging to R31, suggesting that R31 was no longer present in the banana rhizosphere 1 year after application (Figure 1G–J). The most abundant phylum in groups 2019‐CK, 2020‐CK and 2019‐T was Proteobacteria, with a relative abundance of over 30% (Figure 1I,J). In the R31‐treated group in 2020, Actinobacteria (37.5%) were more abundant than Proteobacteria (25.0%). At the genus level, *Streptacidiphilus* was the most abundant in all samples (Figure 1H). Notably, samples in group 2020‐T harbored higher relative levels of *Streptacidiphilus*, *Burkholderia*, and *Streptomyces* than did other groups (Tables S9 and S10).

### Co‐occurrence networks within banana rhizosphere bacterial communities induced by R31

**FIGURE 2:**
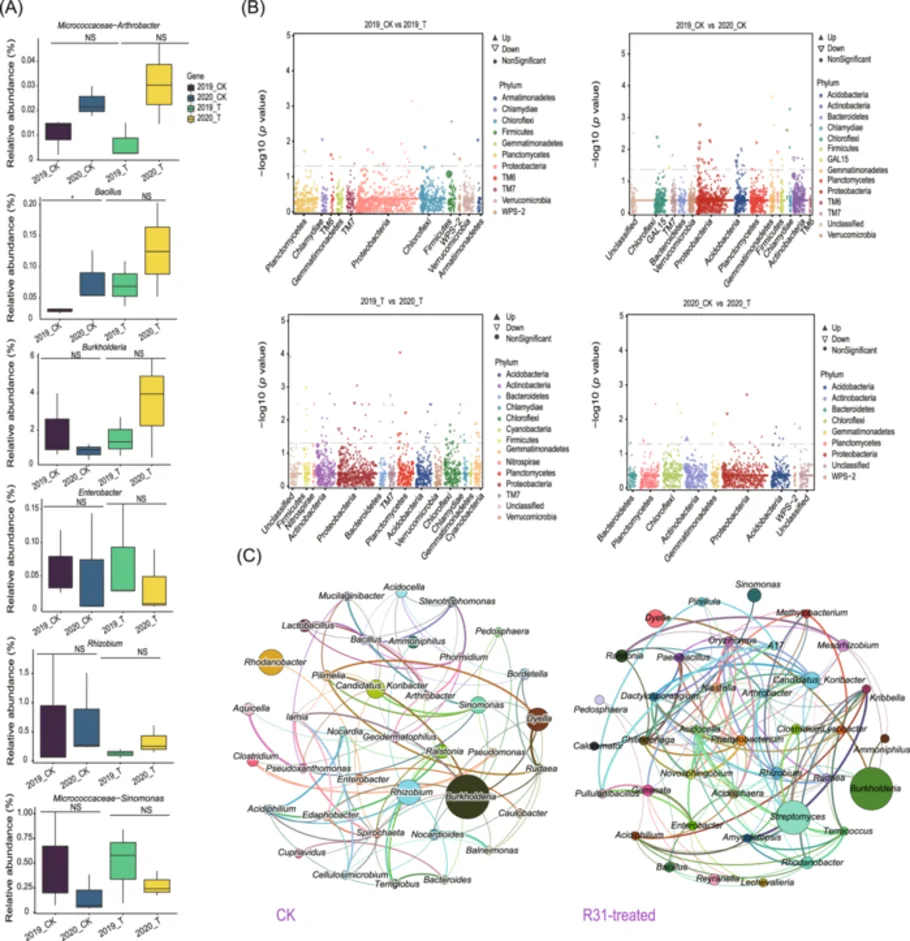
The community succession of core biocontrol genera in different groups based on 16S rDNA sequencing. (A) Relative abundance of the core biocontrol bacteria *Micrococcaceae*‐*Arthrobacter*, *Bacillus*, *Burkholderia*, *Enterobacter*, *Rhizobium*, and *Micrococcaceae*‐*Sinomonas*. NS, No significant difference; * *p* < 0.05 (Nonparametric tests). (B) Community structure in healthy soil and R31‐treated soil. (C) Microbial interaction networks constructed for the core biocontrol genera and significantly interacting genera. Interactions in control samples and R31‐treated samples. The circles represent bacterial taxa and the sizes of the circles represent their abundance. The thickness of the lines represents the strength of the interaction correlations.

Because the composition of bacterial communities varied between 2019 and 2020, the core genera (present in all samples) of R31‐treated and control samples were compared. A total of 76 core genera in the control group, 71 in the R31‐treated group, with 61 shared core genera between the groups (Figures S5 and S6) were detected. Notably, six of the shared core genera (*Arthrobacter*, *Bacillus*, *Burkholderia*, *Enterobacter*, *Rhizobium*, and *Sinomonas*) contained biocontrol species whose function have previously been experimentally validated. The relative abundance of these six genera did not significantly change between 2019 and 2020 in either group, except for *Bacillus* in the control group, with a significantly higher relative abundance in 2020 than in 2019 (Figure 2A,B).

Then, an interactional network for these biocontrol genera and other bacterial genera was constructed, using a significant interaction threshold set to absolute *R* > 0.8 and adjusted *p* < 0.05 (Figure 2C). The core biocontrol genera displayed significant interactions with 31 other bacterial genera in the control group, of which 19 genera were core genera for the control group (Table S11). In the R31‐treated group, 32 bacterial genera, including 26 core genera, significantly interacted with the core biocontrol genera (Table S12). For the core biocontrol genera themselves, *Bacillus*, *Arthrobacter*, *Rhizobium*, and *Burkholderia* showed direct or indirect co‐occurrence with each other in both groups (Figure 2C and Figure S7). In both groups, we also saw the co‐occurrence of *Burkholderia*‐*Dyella* and *Arthrobacter*‐*Ralstonia*, and the mutual exclusion of *Arthrobacter* and *Acidocella*. However, the interaction network of the *Enterobacter* and *Sinomonas* genera was apparently different between the R31‐treated and control groups. Indeed, *Sinomonas* indirectly co‐occurred, whereas Enterobacter showed mutual exclusion with *Bacillus*, *Arthrobacter*, Rhizobium, and *Burkholderia* in the control group (Figure 2C). In the R31‐treated group, *Enterobacter* and *Sinomonas* each formed an interaction network separate from the network comprising *Bacillus*, *Arthrobacter*, *Rhizobium*, and *Burkholderia* (Figure 2C).

### Community succession of suppressive and induced soil

**FIGURE 3:**
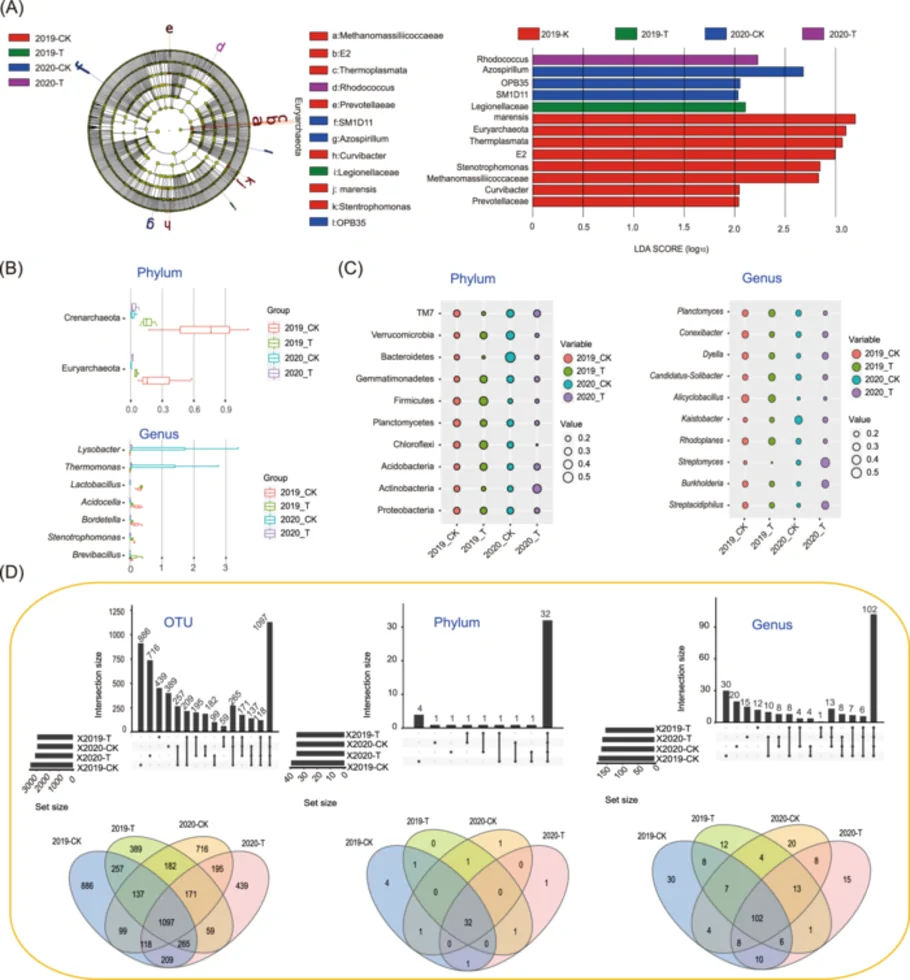
The community succession of the rhizosphere soil bacterial communities in control and *B*. *subtilis* R31‐treated groups. (A) Bacterial biomarkers of different treatments based on LEfSe analysis. The different colors represent the different treatments, and the circles go from inside to outside for the door and genus. The Kruskal–Walis test analysis showed that taxa color‐coded in the evolutionary map had significantly higher relative abundance across treatments. Genera with *p* < 0.05, log LDA scores greater than 3.5, and relative abundance less than 0.1% were not included. (B) According to the Welchs't test, the predominant bacteria (genus level) differed significantly between control and R31‐treated groups. A *p* < 0.05 (or 0.01) was used as the threshold for the results, and smaller *p* values indicated more significant differences. The vertical axis represents the name of distinct phylum and species, the horizontal represents the abundance difference, and the error bar shows the range of the difference in the 95% confidence interval. (C) Indicator analysis based on Bray–Curtis dissimilarity index decomposition between control and R31‐treated groups at the level of the grouping phylum and species classification. The vertical axis of the bubble plot represents the species, the abscissa represents the group, and the size of the bubble represents the indicator value of each species in the group. Bubble colors indicate grouping. (D) Venn diagram and upset diagram analysis of community succession between the control and R31‐treated groups. In Venn diagram, circles represent different groups, overlapping parts between circles represent the number of common OTU, phylum or genus, and nonoverlapping parts represent the number of unique OTU, phylum or genus.

One of the essential features of a microbial community is its dynamics [18, 19] because a community is often in a state of constant change and development [9, 20]. Following treatment with R31 in 2019, the community succession in suppressive and R31‐treated soil from 2019 to 2020 was investigated. Compared to the 2019‐CK group, the relative abundance of Proteobacteria was significantly greater in the 2019‐T group, which had the largest number of OTUs (eight) (Figures 2B and 3A–C). Chlamydiae, Chloroflexi, WPS‐2, Armatimonadetes, and other phyla only had one OTU each with a significantly lower abundance, and the remaining OTUs showed no significant differences in abundance (Figure 3A and Table S13). Compared to the 2019‐CK group, the OTU abundance of Proteobacteria was significantly lower in the 2020‐CK group, with a larger number of 10 OTUs in 2019‐T. The phyla Chloroflexi, GAL15, Planctomycetes, Nitrospirae, Crenarchaeota, and Chlamydiae showed only OTUs with a significantly lower abundance. Notably, most OTUs with differential abundance in soil displayed a significant downward trend in the 2020‐CK group compared to the 2019‐CK group (Figure 3B,C, Tables S14 and S15). Compared to the 2019‐T group, there were significantly more OTUs in the 2020‐T group for Nitrospirae, Actinobacteria, Bacteroidetes, TM7, Acidobacteria, Verrucomicrobia, and Cyanobacteria. The number of OTUs was significantly higher, and the differential abundance of Actinobacteria was the highest (9 OTUs), with Acidobacteria being the second highest (8 OTUs). For Proteobacteria, the number of significantly increased OTUs was 11, and the number of significantly decreased OTUs was three, representing the largest number of OTUs with significant differential abundance (Figure 3D and Tables S16–S18). Compared to the 2020‐CK group, there was only one OTU with a significant difference in the abundance of Actinobacteria in the 2020‐T group, and the abundance of this OTU was significantly greater. OTUs with differential abundance in Bacteroidetes, Chloroflexi, Acidobacteria, and WPS‐2 all showed a significant upward trend. Proteobacteria had the highest number of OTUs with differential abundance, with five OTUs, of which three were significantly higher, and two OTUs were significantly lower.

Taken together, the number of OTUs with significant decreases in abundance was higher for Proteobacteria during secondary succession of suppressive soil. In secondary succession of R31‐treated soil, the number of OTUs significantly upregulated by the differential abundance of Actinobacteria was at most nine, with Acidobacteria being the second most, with eight OTUs. The emergence of R31 biocontrol bacteria affected the succession of suppressive soil, as reflected in the microbial community of Actinobacteria, with one significantly greater OTU. Interestingly, the abundance of Actinobacteria and Firmicutes phyla was reported to be higher in healthy rhizosphere soil than in diseased rhizosphere soil [21].

### Functional properties of the communities related to biocontrol bacteria

The functional properties of bacterial communities were explored with the software Phylogenetic Investigation of Communities by Reconstruction of Unobserved States (PICRUSt2). Then, the contributions of core biocontrol genera and their interacting genera to the abundance of metabolic pathways were estimated based on Spearman correlation coefficients. Accordingly, *Burkholderia* showed a significant and positive correlation with membrane transport and energy metabolism pathways in the control group (*p* < 0.05; Figure S8A,B). There was a similar pattern in the R31‐treated group, although it did not reach significance (Figures S6–S9 and Tables S19–S22). *Burkholderia* also showed a significant but negative correlation with folding, sorting and degradation pathways in both groups. *Rhizobium* showed a significant and positive correlation with pathways related to terpenoid and polyketide metabolism, and a significant negative correlation to pathways related to glycan biosynthesis/metabolism and environmental adaptation in the R31‐treated group. There was a similar trend in the control group, but it did not reach significance. *Sinomonas* was significantly correlated with multiple pathways in the control group but not in the R31‐treated group, and *Bacillus* and *Enterobacter* did not significantly contribute to any pathways in either group. Among the other genera that interacted with core biocontrol genera, *Candidatus* and *Koribacter* showed significant positive correlations with the metabolism of terpenoids and polyketides and the degradation of xenobiotics, and significant negative correlations with glycan biosynthesis and metabolism pathways (Figure S8).

### Isolates from different habitats, their production of enzymes and metabolites, and their in vitro antagonism

**FIGURE 4:**
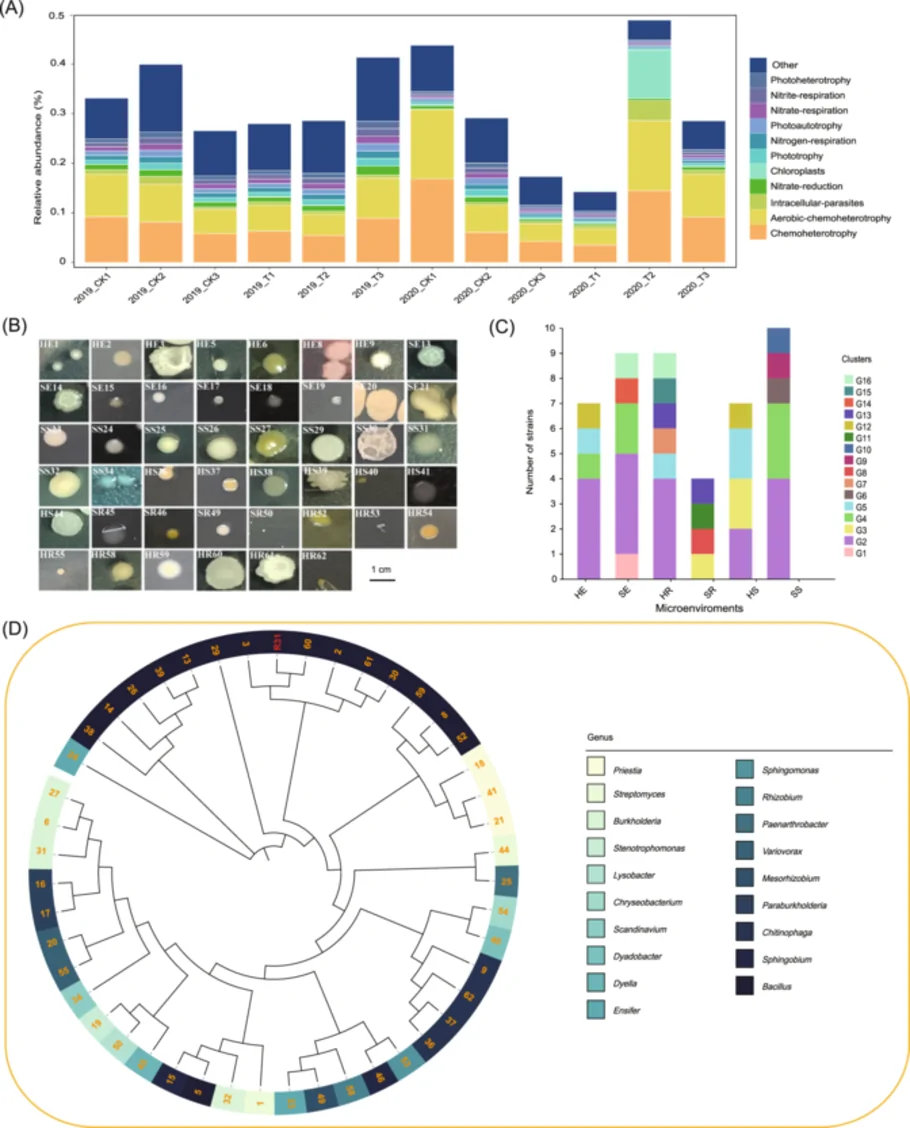
Identification and phylogenetic relationships of R31 and 46 strains isolated from soil. (A) Stacked plot of relative abundance of FAPROTAX functional classification of the control and B31‐treated groups. Stacked plots show different samples on the horizontal axis, and columns of different colors indicate the relative abundance of different ecological functions. (B) Morphologies of single colonies of bacterial isolates from six microenvironments of healthy banana plants and banana plants treated with R31. HE 1–9 are endophytic bacteria in the roots of banana plants treated with *B. subtilis* R31. SE 13–21 are endophytic bacteria in the roots of naturally healthy banana plants. SS 23–34 are banana plant rhizosphere bacteria for natural health. HS 36–44 are bacteria in rhizosphere soil of banana plants treated with *B. subtilis* R31. SR 45–50 are exogenous bacteria from the roots of naturally healthy banana plants. HR 52–62 are root ectomyophytes of banana plants treated with *B. subtilis* R31. Scale bar, 1 cm. (C) Forty‐six bacterial isolates were subjected to ARDRA and 16S rDNA gene sequencing. 16S rDNA gene sequencing resulted in 16 clusters (Tables S4–S2 and Figure S1A). (D) Sequences of R31 and 46 strains isolated from soil were aligned using MAFFT, and then analyzed using the Neighbor‐Joining method to determine phylogenetic relationships.

Overall, the core genera in the naturally formed suppressive soil and R31‐induced suppressive soil were highly consistent. According to the “amplication‐selection” assembly model of the rhizosphere microbiome and the concept of “metropolis,” microorganisms are thought to be gradually selected in the soil, rhizosphere, and internal roots to form plant rhizosphere‐specific microbial communities [17]. Based on the Greengenes database, we used Bugbase for the phenotypic prediction of communities. By integrating the gene information of IMG, KEGG, and PATRIC databases, they were divided into seven main types including Gram Positive, Gram Negative, Biofilm Forming, Pathogenic, Mobile Element Containing, Oxygen Utilizing (including Aerobic type: Aerobic, Anaerobic type: Anaerobic, facultative anaerobic: Facultatively anaerobic) and Oxidative Stress Tolerant (Oxidative Stress Tolerant). We noticed a similar phenotypic prediction and phenotypic contribution in the suppressive soil and R31‐induced (Figure 4A, Figures S10–S11, and Tables S23–S25). To reveal the regularity of microbial communities selected in the soil, rhizosphere, and internal roots of naturally suppressive soil and R31‐induced suppressive soil, a total of 46 bacterial isolates from six microenvironments of naturally suppressive soil and R31‐induced suppressive soil were obtained using a culture‐dependent method: the exterior and interior of healthy banana plants roots (SR and SE, respectively); the exterior and interior of banana plants roots treated with R31 (HR and HE, respectively); the surrounding bulk soils of healthy banana plants and banana plants treated with R31 (SS and HS, respectively) (Figure 4B and Table S26). The numbers of isolates retrieved from HR (nine), HE (seven), and HS (seven) were comparable to those from SR (four), SE (nine), and SS (ten), respectively (Figure 4C). Among the six microenvironments, SE and HR had the highest and lowest bacterial population densities (128.3 × 10^4^ colony‐forming units [cfus]/g roots and 46.7 × 10^4^ cfus/g roots, respectively). We subjected 46 bacterial isolates to amplified ribosomal DNA restriction analysis (ARDRA) and 16S rDNA gene sequencing. 16S rDNA gene sequencing resulted in 16 clusters (Figure 4C,D). Of the 46 isolates, 34 (73.9%) produced protease, five (10.9%) produced chitinase, 38 (82.6%) produced cellulase, 22 (47.8%) produced *β*‐1, 3‐glucanase, and 45 (97.8%) produced the auxin molecule IAA (Figures S12–S15 and Table S27).

**FIGURE 5:**
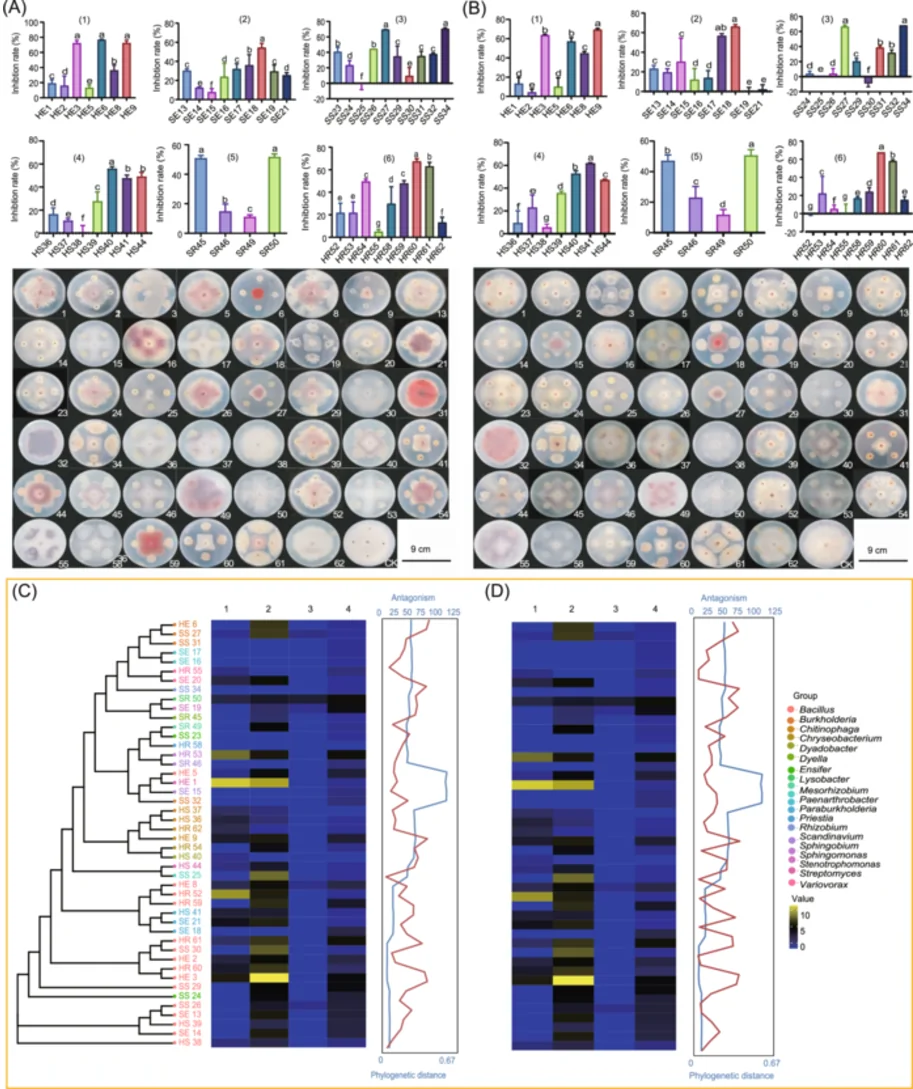
Antagonism, phylogenetic distance and enzyme activity of 46 bacterial isolates. (A) The inhibition rate of BACs against FOC 004: The bar graphs (1)–(6) represent the inhibition rate of BACs (Beneficial strains) from different habitats against FOC 004 in different habitats. Different lowercase letters indicate significant differences between strains (*p* < 0.05). Data analysis was performed by SPSS (methods using LSD) and Duncan). Scale bar, 9 cm. (B) The inhibition rate of BACs against FOC 009. (C, D) The Neighbor‐Joining trees to the left of the heatmaps were reconstructed based on the 16S rDNA sequences of the 46 isolated strains after alignment using MAFFT. The heatmaps indicate *β*‐1, 3‐glucanase activity (1), protease activity (2), chitinase activity (3), and cellulase activity (4) of the 46 bacterial isolates. The red curves in the boxes to the right show the antagonistic phenotypes of FOC004 (C) and FOC009 (D), and blue curve shows phylogenetic distance between R31 and each of the isolated strains.

The present study demonstrated that antagonism tended to be positively correlated with phylogenetic distance from the genus *Bacillus* [3, 22]. The investigation of enzyme production activities, antagonism, and phylogenetic distances from R31 for each of the 46 bacterial isolates was performed (Figure 5). Almost strains in the phylogenetic tree had strong abilities to produce protease, especially strain HE1 (*Streptomyces chrysomallus* from the Streptomycetaceae) and strain HE3 (*B. velezensis* from the Bacillaceae). Strain HE1 produced more *β*‐glucosidase activity but was phylogenetically distant from the other strains including strain HR52 (*B. proteolyticus* of the Bacillaceae) and strain HR53 (*Sphingomonas jeddahensis* of the Sphingomonadaceae). We also observed that strain SR50 (*Lysobacter enzymogenes* of the Xanthomonadaceae) shows good enzyme production for these four enzymes, especially cellulase. Interestingly, strain SR50 also showed antagonistic activities against FOC004 and FOC009 (Figure 5A,B and Table S28). The strains with close phylogenetic distance to R31 had weak *β*‐glucosidase production, strong protease production, weak chitinase production, and average cellulase production. In addition, regardless of the phylogenetic distance, the ability of the strains isolated from the soil to produce chitinase was weak, and their ability to produce cellulase was average. Strains SE16, SE17, SS31, SS32, SE15, SS23, and SR46 had similar enzyme production abilities, as well as weak antagonistic activity. Based on these results, we conclude that the antagonistic activity is more closely related to the enzyme production activity of the strains than to their phylogenetic distances (Figure 5C,D).

### Kin discrimination between R31 and banana rhizosphere microbial flora

**FIGURE 6:**
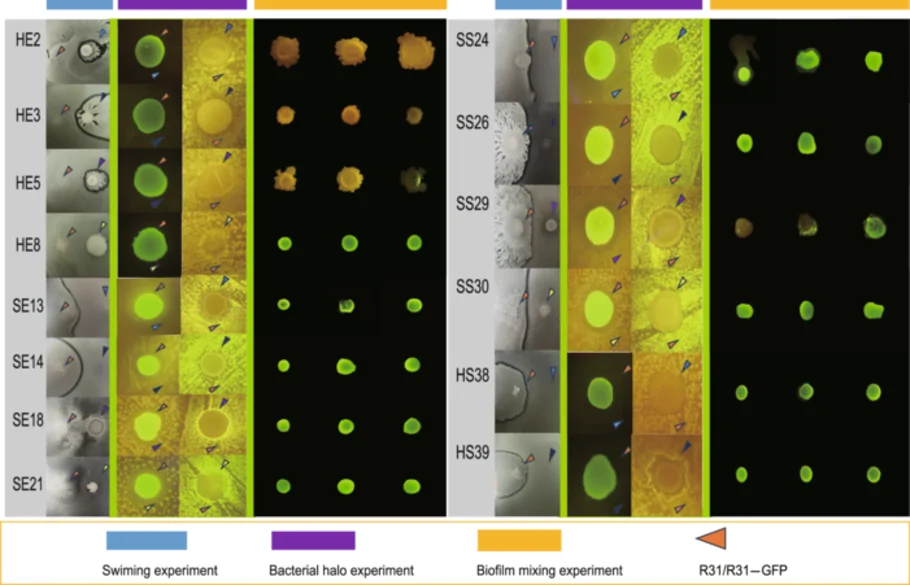
Kin discrimination between *B. subtilis* R31 and endophytic bacteria in the roots of banana plants in natural healthy plots. Swimming experiment: Morphological characteristics of the swarming experiment using R31 and the indicated strains. Bacterial halo experiment: Morphological characterization of halo measurement using GFP‐Labeled R31 and the indicated strain, observed using a Leica Laser microscope at 488/510 nm (fluorescence mode). Biofilm mixing experiment: Biofilm mixing experiment of R31 and the indicated strain using a Leica Laser microscope at 488 nm (fluorescence mode). GFP‐Labeled *B. subtilis* R31 and the indicated strain observed in a 1:1 inoculation ratio. GFP‐Labeled *B. subtilis* R31 and the indicated strain observed in a 1:2 inoculation ratio. GFP‐Labeled *B. subtilis* R31 and the indicated strain observed in a 2:1 inoculation ratio. The orange arrow represents GFP‐labeled *B. subtilis* R31; arrows in other colors represent the tested strains.

A negative correlation was previously reported between antagonism and phylogenetic distance [23]. A positive relationship was also identified between antagonistic interaction and phylogenetic dissimilarity in *B. subtilis* [24]. Kin discrimination can help biological systems direct cooperative behavior toward their relatives two forms of kin recognition and non‐kin exclusion [9, 24]. The Colony‐merger incompatibility phenomenon is an important phenotype for the identification of bacterial relatives during the process of kin enrichment, including the fusion, hemi‐fusion, and boundary phenomena [25]. Here, we investigated the ability of R31 and isolated bacteria to discriminate kin from nonkin in the context of swarming, production of mixed‐strain biofilms (Figure 6), and antagonistic activity (the strains with distant phylogenetic to R31; Figures S16–S21).

In a swarming experiment, 4 strains came into contact with R31: HE8, SE18, SE21, and HS38. Two strains, SE14 and SS30, showed hemi‐fusion with R31. A total of 8 strains had boundary phenomenon with R31: HE2, HE3, HE5, SE13, SS24, SS26, SS29, and HS39. In a halo bioassay, the results showed that coated R31 expressing the green fluorescent protein (GFP) for ease of identification onto MSgg plates, before examining fusion between R31‐GFP and the tested *Bacillus*. On MSgg plates, R31‐GFP fused with SE13 in one colony, and had boundary phenomenon with 13 other strains (Figure 6).

In a biofilm mixing experiment, *B. subtilis* R31‐GFP co‐existed with strains HE8, HS38, HS39, SE13, SE14, SE18, SE21, SS24, SS26, SS30, and SS24 co‐existed when mixed in the 1:1, 1:2, and 2:1; R31‐GFP co‐existed with HE5 and HR61 strains at a 2:1 ratio, but only one strain survived at the ratios of 1:1 and 1:2; R31‐GFP could not survive. The strains HE2 and HE3 remained unmixed when they were in the coculture system. Among them, HE2, HE3, and HE5 strains survived when cocultured with R31‐GFP, but the other 11 strains did not (Figure 6). Bacterial antagonism tests showed no significant effect on the colony diameter of *B. subtilis* R31 as the inoculation distance to the tested strains during the process of kin enrichment.

### Functional analysis of microbial flora isolated from banana rhizosphere soil

**FIGURE 7:**
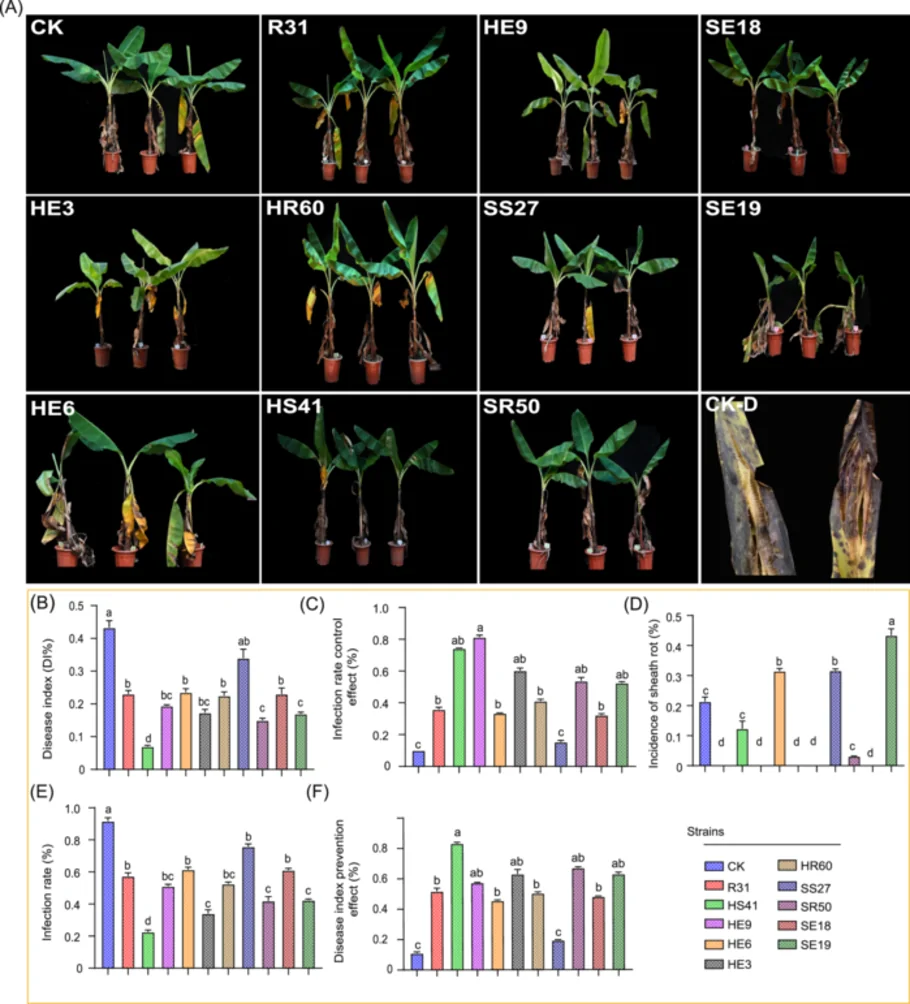
Biocontrol effects of target strains on Fusarium wilt in potted banana plants. (A) The pot plant picture on the top indicated: Biological control effects of CK: Control; R31: *B*. *subtilis* R31; HS41: *Bacterium* sp. HS41; HE9: *Chitinophaga* sp. HE9; HE6: *Burkholderia* sp.HE6; HE3: *B*. *amyloliquefaciens* HE3; HR60: *B*. *amyloliquefaciens* HR60; SS27: *Burkholderia* sp. SS27; SR50: *Lysobacter* sp. SR50; SE18: *B*. *megaterium* SE18; SE19. *Stenotrophomonas panacihumi* SE19; CK‐D: Control after dissection. Bar graph on the bottom indicated: (B) the disease index, (C) infection rate control effect (%), (D) incidence of sheath rot (%), (E) infection rate (%), and (F) disease index prevention effect (%) of the tested strains. Error bars represent the mean ± SD of the diameters. Different lowercase letters indicate significant differences (Duncan's new multiple range test, *p* < 0.05).

Based on the above work and our analysis, we identified potential target biocontrol strains for tests in pot experiments. After calculating the disease index of each treatment [26], we determined that symptoms in the banana seedlings treated with strains HE3, HE9, SE19, HS41, and SR50 were significantly mitigated compared to those seen in the control group. Indeed, the infection rates of pathogenic bacteria were below 50%, and the disease index control efficiencies were over 60%, demonstrating a beneficial effect of treatment with these strains. All the indexes of banana seedlings treated with strain HS41, HE3, HE9 SR50, and SE19 were significantly better than those treated with other strains (Table S29 and Figure 7A–F). During the disease diagnosis, many plants were severely infected with sheath rot despite not having been inoculated with the pathogen *F. oxysporum* f. sp. *cubense* FOC004 and FOC009 causing banana sheath rot, making it necessary to consider that influence. In particular, many banana plants treated with strains HE6, HS41, SE19, SS27, and SR50 showed cross‐infection symptoms of sheath rot with the rate of 30%, 10%, 43.3%, 30%, and 3.33%, respectively (Table S29). Synthetically, the strains HE3 and HE9 show potential for better than HS41, and SR50 better than SE19. These results showed that the control effect of R31‐induced soil bacteria is better than that of naturally suppressive soil bacteria. Especially, the bacteria isolated from the inside of the root system (R31 application) shows better‐integrated control effect compared to those from the rhizosphere (R31 application), and the bacteria isolated from rhizosphere (healthy soil) shows better‐integrated control effect compared to those from the inside of the root system (healthy soil).

## 3 DISCUSSION

The microbial structure and function in rhizosphere soil or roots are not static but in a state of succession [27], and the assembly of root‐associated microbiome is also affected by environmental factors. Several studies have shown that strains introduced to manipulate microbiomes are eliminated in soils, whereas other studies have reported that the application of plant growth‐promoting bacteria significantly improves plant growth [15, 28, 29, 30]. This contradiction suggests the need for a deeper understanding of the mechanisms underlying the microbe‐induced distribution and succession of microorganisms in soils [31]. Here, for the first time, we investigated the secondary succession of microorganisms in rhizosphere soil with continuous cropping of banana for more than 5 years compared with suppressive soil effected by applying biocontrol R31 bacteria. We observed a convergent secondary succession of the rhizosphere bacterial community during naturally suppressive soil and R31‐induced suppressive soil, which exhibited consistent co‐occurrence of *Burkholderia*‐*Dyella* and *Arthrobacter*‐*Ralstonia* as the core genera. We provided the evidence that the common disease‐suppressing soil induced by biocontrol bacteria can be transferred to specialized types of soil disease inhibition.

Microorganisms that cause disease suppression can be divided into normal and specialized types [2]. Soil microorganisms in the common disease‐suppressing soil can compete with soil‐borne disease agents for nutrients, infection sites, or colonization of host plants. The common disease‐suppressing soil cannot be transferred to different soils. In the case of specialized microorganisms, the production of antibiotics or other antimicrobial substances plays a role in disease suppression, with transferability being the main characteristics of disease suppression. The specific disease suppression, also known as induction type disease suppression (induced suppression), usually through certain agronomic measures regulation can be formed [2]. We analyzed core microbiota, biocontrol genera abundance, species correlations, microbial interaction networks as related to biocontrol bacteria, and potential functions of key species. The biocontrol bacterium *B. subtilis* R31, to some extent, enhanced the association of *Streptomyces* with the rhizosphere core genera and weakened the association of *Burkholderia* with the functional pathways membrane transport and energy metabolism in the community. R31 significantly enhanced a positive correlation of *Rhizobium* with the metabolism of terpenoids and polyketides pathway in the treated group, with glycan biosynthesis and metabolism, and environmental adaptation pathway is significantly negative correlation. 46 bacterial isolates from six microenvironments of naturally suppressive soil and R31‐induced suppressive soil were isolated using a culture‐dependent method and examined for their ability to produce IAA, protease, cellulase, chitinase, and *β*‐1, 3‐glucanase activity, and for kin discrimination and antagonism in vitro and in vivo. The results indicated that R31 is likely to prevent and control banana Fusarium wilt through regulating the banana rhizosphere microorganism flora structure (especially increase the microbial abundance of Actinomyces), function, which help potential biocontrol bacteria grow into the plant roots.

**FIGURE 8:**
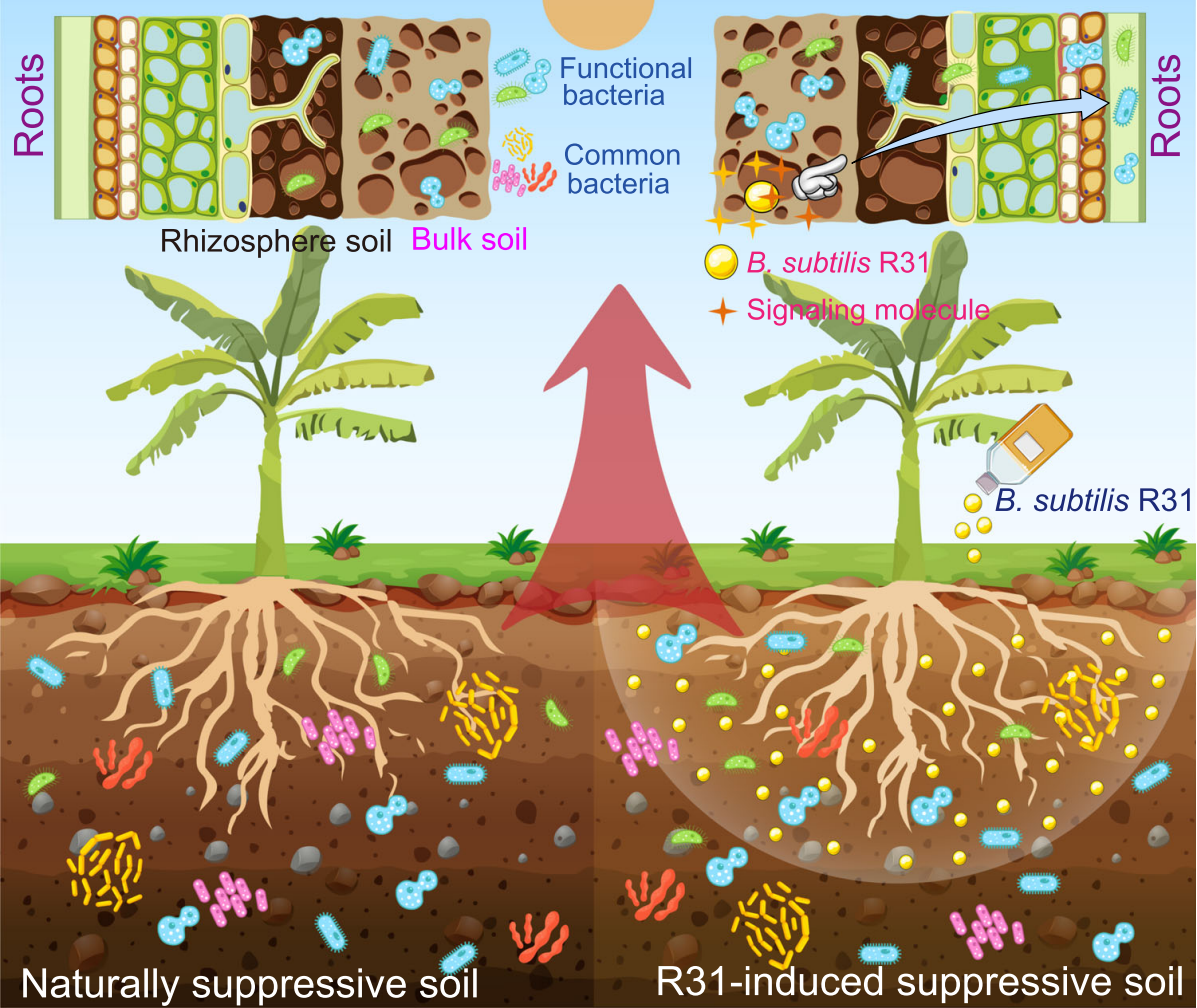
Model showing how *B. subtilis* R31 promotes the control of Fusarium wilt by influencing the rhizosphere strains colonizing banana plants. *B. subtilis* R31 (yellow ball) regulates the structure and function of the banana rhizosphere microorganism flora, to prevent and control banana Fusarium wilt. During the promotion of root hair growth of banana roots, R31 colonizes root hairs; through the action of cellulose, chitinase, and other soluble secondary metabolites, R31 changes the pores on the surface of the root hair. Using chemical communication (gold aster), R31 induces the functional bacteria to rapidly migrate into the nascent root hair, promoting colonization.

The priority effects theory states that the order in which microbes colonize can determine the ability of beneficial bacteria to resist later pathogen invasion [32]. Preferentially established communities are more stable and resistant to later invaders, and are largely unaffected by later invaders [33]. In general, the kin discrimination experiment of R31 and isolates collected from the Foshan Sanshui location showed that R31 and most of the isolates exhibited boundary phenomena and were distantly related in a swarming assay. Interestingly, R31 was not detected in the rhizosphere during the second year, suggesting its disease‐suppressing effects might be mediated by its initial modulation of the rhizosphere microbial community, which persisted even after R31 disappeared. This highlights a potential “legacy effect” of R31 in disease suppression. In particular, we hypothesize that, during the promotion of hair growth of banana roots, R31 induced functional bacteria to migrate to the nascent root hair tissue through chemical communication, and then formed new colonization patterns (Figure 8). Notably, the disappearance of biocontrol R31 bacteria and the preferential effector of rhizosphere microbiome were most likely not determined by rhizosphere microbial interactions, so clearly more in‐depth studies are needed.

Here, we found that the swarming and biofilm mixing of R31 and tested 46 bacterial isolates did not necessarily closely related phylogenetic distance, but the enzyme production activity was positively correlated with the antagonistic activity against Fusarium wilt. Especially if the bacteria were isolated from the inside of the root system (naturally suppressive soil and R31‐induced suppressive soil) compared to those isolated from the outside. Antagonism tests showed no clear pattern between *B. subtilis* R31 and the 46 tested bacterial isolates. This kind of role‐switching sometimes depends on the contact distance between strains, suggesting that volatile organic compounds and other soluble secondary metabolites might be involved in these microbial interactions. We speculate that there is a complex cooperation between biocontrol strain R31 and the rhizosphere microbiome of banana, including neutralism, commensalism, synergism, mutualism, amensalism, and competition.

Pot experiments showed that potential target strains showed a strong control effect on banana Fusarium wilt caused by FOC004 and FOC009 without a clear pattern. However, endophytic bacteria in roots exhibited better performance than bacteria in rhizosphere soil in terms of their effectiveness in Fusarium wilt prevention and control. But it should be noted that the banana plants treated with single more likely showed cross infection symptoms of sheath rot. Therefore, strains with biocontrol potential are more likely to exhibit stable colonization of plants before they can stimulate disease resistance and growth promotion. There are also many microorganisms that induce no resistance individually. Thus, we propose that the expulsion of pathogens in soil is not due to a single strain, but more likely to the synergistic actions of several organisms. Increasing attention has been paid to the use of synthetic microbiota to achieve the disease‐resistance and growth‐promoting functions of biocontrol bacteria [34, 35]. However, there is still a lack of scientific guidance on how to construct such synthetic bacterial communities and how to select potential biocontrol bacteria. Overall, our study revealed that selecting bacterial isolates that secrete high levels of extracellular enzymes and have a strong ability to colonize potential biocontrol bacteria may be an effective strategy for selecting potential biocontrol bacteria and constructing a beneficial synthetic bacterial community. Further research is needed to explore the molecular mechanisms underlying the interactions between R31 and other rhizosphere microbes, including the role of volatile organic compounds and secondary metabolites in mediating microbial community dynamics. Additionally, long‐term field studies are essential to confirm the persistence and transferability of R31‐induced disease suppression.

## 4 CONCLUSION

The common disease‐suppressing soil induced by *B. subtilis* R31 can be transferred to specialized types of soil disease inhibition. The *B. subtilis* R31 enhanced the association of *Streptomyces* with the rhizosphere core genera and weakened the association of *Burkholderia* with the functional pathways of membrane transport and energy metabolism in the community. The *B. subtilis* R31 induced potential biocontrol bacteria to grow into the plant roots. The antagonistic activity of soil microorganisms is more closely related to the enzyme production activity of the strains than to their phylogenetic distances. In all, as an important agronomic measure, the application of *B. subtilis* R31 can provide the convergent secondary succession of the rhizosphere bacterial community in naturally suppressive soil and R31‐induced suppressive soil.

## 5 METHODS

### Field experiment description and sampling

To examine the biocontrol of banana Fusarium wilt disease, plants were treated with *B. subtilis* R31. This work was carried out in a banana orchard in Sanshui, Foshan, China, planted with Brazilian banana (*Musa nana* Lour.) for more than 5 years and treated with *B. subtilis* R31 in March 2019 [36]. Briefly, a single R31 colony was selected, and placed into 100 mL nutrient broth (NB) medium (consisting of Beef extract, 3.0 g; peptone,10 g; NaCl, 5 g; 15 g agar in 1 L of distilled water; pH 7.2), and cultured overnight at 37°C with shaking at 180 rpm. Then, 12 mL of the bacterial suspension was added to 200 mL of NB broth and cultured for 24 h at 180 rpm and 37°C. The number of cells in the broth was counted, and broths containing different individual strains were mixed with the R31 broth in equal proportions so that the titer of the mixed broth was 1 × 10^8^ cfu/mL. Then, 100 mL of the mixed broth was poured around the roots of each banana seedling, and a corresponding volume of water was sprayed onto other plants as a control. Following the application of R31, rhizosphere soil samples and roots were collected in September 2019 and 2020 from banana plants and from healthy banana plants that had been planted 5 years earlier. The root and rhizosphere soil samples were placed in numbered bags and transported back to the laboratory in ice boxes for storage at 4°C.

### DNA extraction

Bacterial strains were isolated and purified from soil samples taken in the banana orchard in Sanshui, Foshan, China. The strains were individually inoculated into LB liquid culture medium (50 mL), grown for 24 h at 30°C with shaking at 200 rpm, and then the bacteria were collected by centrifugation at 10,000 rpm for 10 min. A Minibest Genomic DNA Extraction Kit 3.0 (TaKaRa) was used to extract the genomic DNA from bacterial strains according to the manufacturer's instructions.

### Sequencing and bioinformatic analysis

The V3–V4 hypervariable region of 16S rDNA was amplified using 20–30 ng of genomic DNA using pairs of primers synthesized by Gene Denovo Ltd. (Guangzhou, China). The forward and reverse primers for 16S rDNA amplification were 5′‐CCTACGGGNGGCWGCAG‐3′ and 5′‐GGACTACHVGGGTATCTAAT‐3′, respectively. Index adapters were connected to the ends of amplicons to generate indexed libraries [37]. Quality control of PCR products with index adapters was performed using a Qubit 3.0 Fluorometer (Invitrogen) and adjusted to 10 nmol for high‐throughput sequencing. The DNA libraries were loaded onto an Illumina MiSeq instrument according to the manufacturer's instructions, and sequencing was performed using the paired‐end 250‐bp mode. FastP software (version 0.18.0) was used to trim raw reads and generate clean reads. The clean reads were assembled into raw tags using FLASH software (version 1.2.11). After the removal of chimeric reads, the effective tags were clustered into OTUs using the USEARCH software (version 9.2.64). The OTUs were annotated using the SILVA database v132 for 16S rDNA [38]. Singletons across all samples were removed from the subsequent analyses. Microbiome interaction networks were visualized using Cytoscape v3.8.2 [39]. Functional annotations of the microbiome were further obtained based on the Kyoto Encyclopedia of Genes and Genomes (KEGG) pathway analysis using PICRUSt2 [40].

Alpha diversity indices, including the observed species richness, Shannon, and Simpson indices for diversity, were calculated in QIIME2 and visualized using R software v3.6.3. One‐way analysis of variance (ANOVA) followed by Tukey's honestly significant difference to assess the differences among the different groups. Statistical significance was set at *p* ≤ 0.05. Dissimilarities in taxonomic diversity among samples were visualized using beta‐diversity of PCoA with a Bray–Curtis distance matrix of taxon relative abundances using the VEGAN v2.5‐7 package in R, and pairwise PERMANOVA was performed to estimate the statistical significance between different groups. Core genera were determined using the “microbiome” and “dplyr” packages in R [38]. The co‐occurrence networks of microbiota were estimated at the genus level according to pairwise associations using Spearman's correlation coefficients. Significant interactions were those that satisfied the thresholds of absolute *R* > 0.8 and *p* < 0.05 (Benjamini–Hochberg adjustment). *T*‐test analysis was used to assess differences between groups (|FoldChange| > 2, *p* < 0.05), with significant difference OTU of phylum and contains all the OTU drawing, using R package ggplot2 [41]. The phylum was used as the *x*‐axis and OTU abundance as the *y*‐axis to draw a Manhattan map of soil differential OTUs [41]. 16S rDNA gene sequencing results of the 46 isolates were confirmed using Basic Local Alignment Search Tool (BLAST) (https://blast.ncbi.nlm.nih.gov/Blast.cgi). MAFFT was used to align the sequence data, and the sequences were analyzed by reconstructing a phylogenetic tree using the neighbor‐joining (NJ) method.

### Isolation of bacterial isolates

Roots, bulk soil, and rhizosphere soil of healthy banana fields were collected in the banana orchard located in Sanshui, Foshan. Roots and rhizosphere soil from banana fields to which *B. subtilis* R31 had been applied were collected, immediately transferred individually into numbered plastic bags, and transported to the laboratory for isolation of bacteria. Samples of banana roots were soaked in 1% (w/v) sodium hypochlorite for 5 min, in 70% (v/v) ethanol for 1–2 min, and washed three times with sterile water. One hundred microliters of the final wash was applied to a R_2_A (consisting of Yeast powder, 0.5 g; Glucose 0.5 g; Casein peptone, 0.25 g; C_3_H_3_NaO_3_, 0.3 g; Meat peptone, 0.25 g; Hydrolyzed casein, 0.5 g; Starch, 0.5 g; MgSO_4_.7H_2_O, 0.024 g; K_2_HPO_4_, 0.3 g; 15 g agar in 1 L of distilled water; pH 7.2) plate and the surface was considered clean and sterile if there was no bacterial growth after 48 h. Three grams of a surface‐sterilized sample was placed in a mortar and pestle, 27 mL of sterile 0.85% (w/v) NaCl was added, and the soft tissue was soaked, ground, and filtered with a gauze filter. This filtrate was then serially diluted tenfold using sterile 0.85% (w/v) NaCl. Then, 100 μL of each of the four dilutions (10^−3^, 10^−4^, 10^−5^, and 10^−6^) was applied to R_2_A plates, with three replicates each, and plates were incubated upside down at 30°C for 48 h. Plates with 30–300 colonies were selected, and colonies of different sizes and morphologies on the plates were selected for isolation and purification on LB medium. The strains were preserved in 40% (v/v) glycerol and stored in an ultra‐low temperature refrigerator at −80°C.

For strains isolated and purified from banana rhizosphere soil samples, 3 g of a banana rhizosphere soil sample was placed in a sterile conical flask, 27 mL of sterile 0.85% (w/v) NaCl, and 3 g of sterilized glass beads were added, flasks were shaken for 30 min at 120–150 rpm, and the suspensions were allowed to settle for 5 min to obtain a 10^−1^ dilution. Serial dilutions were generated with 100 μL of this initial suspension with 900 μL sterile 0.85% (w/v) NaCl to obtain 10^−2^, 10^−3^, 10^−4^, 10^−5^, and 10^−6^ suspensions. Then 100 μL of the 10^−3^, 10^−4^, 10^−5^, and 10^−6^ dilutions were spread onto R_2_A medium, with three replicates each, and plates were incubated upside down at 30°C for 48 h. Plates with 30–300 colonies were selected, and colonies of different sizes and morphologies were selected for isolation and purification on LB medium. The strains were stored in a final concentration of 40% (v/v) glycerol in an ultra‐low temperature refrigerator at −80°C. The method for isolating bacteria from the bulk soil was similar to that used for isolating rhizosphere soil bacteria.

### Detection of the activities of hydrolases and metabolites from bacterial isolates

All 46 bacterial isolates obtained were examined for their ability to produce extracellular hydrolytic enzymes and IAA. Protease activity, as indicated by casein degradation, was determined based on the widths of cleared zones surrounding colonies grown on skimmed milk agar (Skim milk powder 6.4 g (Ding chang sheng biotechnology co., LTD., Beijing DH220‐3, 100 g, RT), add to the pure water, blending the capacity to 240 mL). Cellulase activity was evaluated based on the widths of clear zones around colonies grown on chitin‐agar plates (NH_4_H_2_PO_4_ (Shanghai Spike Testing Company, 500 g, AR) 1.0 g, KCl (Shanghai Spike Testing Company, 500 g, AR) 0.2 g, MgSO_4_• 7H_2_O (Xilong Chemical Co., LTD., 500 g, AR) 0.2 g, Take colloidal chitin in 1% (w/v) with the capacity to 1000 mL, the deionized water pH = 7.0, 20 g agar). *β*‐1, 3‐glucanase activity was determined using *β*‐1,3‐glucanase‐agar plates (*β*‐1,3‐dextran (McLean, 70%), 5 g, 0.1 g, TSB (pancreatic dairy soy peptone liquid medium, Guangdong ring microbial kay technology co., LTD., 250 g) 0.4 g, agar 1.6 g, 4 g/L of Congo red (Shanghai Sanai Reagent Co., LTD., 25 g), 1 mL, blending the capacity to 100 mL), and IAA was quantified with Ehrlich's reagent (Dimethyl amine benzaldehyde (Tianjin days new fine chemicals development center, 25 g, AR) 8 g, blending to 760 mL with 95% alcohol constant and 160 mL of concentrated hydrochloric acid). In these assays, each isolate was tested in one replicate plate, and each plate consisted of four inoculation replicates, giving a total of four replicates per isolate in each assay [36].

### *In vitro* antagonism assay of the 46 isolates

The banana Fusarium wilt pathogen (*F. oxysporum* f. sp. *cubense*), strains FOC 004 and FOC 009 (isolated from diseased banana plants), were stored at 4°C and inoculated onto potato dextrose agar (PDA) plates (consisting of 200 g potato extract, 1 g peptone, 20 g; sucrose; 20 g agar in 1 L of distilled water; pH 7.0), and then incubated at 30°C in darkness for 7 days. An overnight culture of a selected bacterial isolate grown in LB broth was spotted 2.7 cm from the center of a 9.0‐cm PDA plate (v/v, 1:1, a total of 15 mL per plate), and a 0.5‐cm plug from the leading edge of a culture of the pathogen grown for 7 days at 30°C on PDA medium was placed in the center of the plate. Each plate consisted of four inoculation replicates for each isolate, with a noninoculated PDA plate serving as the control. Each isolate was tested in three replicate plates, giving a total of 12 inoculation replicates per isolate. Plates were incubated at 30°C and scored after 5–7 days by measuring the sizes of the inhibition zones between the edges of the bacterial colony and the fungal mycelium. After the mycelium of the control overgrew the plate, the diameter of the mycelium growth zone was recorded, and the inhibition rate was calculated with the following formula: Inhibition rate (%) = [(ΦCK − Φ0) − (Φtreatment − Φ0)]/(ΦCK − Φ0) ×100, where Φ is the diameter of the mycelium growth zone, ΦCK is the diameter of that of the control, Φ0 is the diameter of the pathogen disc with 5 mm, and Φtreatment is the diameter of the mycelium growth zone for an isolate) [14]. Three replicates were performed for each isolate.

### Evaluation of the 46 isolates with an assignment system

To evaluate the biocontrol potential of all 46 isolates, an assignment system was devised that comprised six parameters: the antagonism of an isolate toward a pathogen, protease activity, chitinase activity, cellulase activity, *β*‐1,3‐glucanase activity, and IAA activity. The activity of an enzyme was based on the size of the hydrolysis ring from each isolate: 0, 1, 2, and 3 stand for hydrolysis ring sizes of 0, 1–3, 3–6, and >6 mm, respectively. Bacterial antagonism was based on the inhibition rate: 0, 1, 2, 3, 4, and 5 represent inhibition rates of 0%, 0–20%, 20–40%, 40–60%, 60–80%, and 80–100%, respectively. IAA production was scored as follows: 0, no red color; 1, light red; 2, red; and 3, dark red [42]. The score for each isolate was the sum of values assigned to the six parameters. Genomic DNA was extracted from each isolate, and purified using a MiNiBEST Genomic DNA Extraction Kit Version.3.0 (TaKaRa Co. Ltd.), the 16S rDNA gene fragments were amplified from genomic DNA using 16S rDNA universal primers [U8‐27(F), 5′‐AGA GTT TGA TCC TGG CTC AG‐3′; and L1492(R), 5′‐ ACG GCT ACC TTG TTA CGA CTT‐3′] for amplified ribosomal DNA restriction analysis (ARDRA) [43]. The 16S rDNA gene amplification products were purified, ligated into the pMD19‐T vector (Transgen Biotechnology Co., Ltd.), and transformed into *Escherichia coli* DH5α (Vazyme), generating positive clones containing plasmids with inserted DNA. Sequencing of 16S rDNA gene fragments was performed at Sangon Biotech Institute, and sequence analysis was conducted using the BLAST from the National Center for Biotechnology Information (NCBI). The evolutionary distance between each sequence and R31 was obtained using MEGAv5.0 [44]. The enzyme activity was plotted against the phylogenetic tree data using the R package ggtree [38]. Subsequently, the R package ggtree was used to map the genus information and phylogenetic tree information of these 46 strains and R31 [45].

### Swarm boundary assay

The tested strains were streaked onto LB plates and activated overnight (first‐level seeds) at 37°C. Bacteria from single colonies were transferred into 5 mL liquid LB medium and cultured for 12–14 h at 37°C and shaking at 180 rpm (second‐level cultures). From 5 mL seed suspension, 1% (v/v) of the bacteria was added to 5 mL liquid LB medium and cultured for 4 h at 37°C and shaking at 180 rpm (third‐level cultures). Suspensions of third‐level cultures were diluted with sterile water to OD_600_ = 0.5. Then, 2 μL of this suspension was inoculated on a 0.5% (w/v) Na^+^ solid plate (consisting of NaCl, 5 g; beef extract, 3 g, bacterial peptone, 10 g; 15 g agar in 1 L of distilled water; pH 7.0), and two types of bacteria were inoculated on medium at a distance of 2 cm apart for testing. The experiment was triplicate. Discard suspension, the plate was placed in an incubator at 37°C for 2 days and the experimental results were recorded by photography. On MSgg solid medium (consisting of K_3_PO_4_.3H_2_O (pH7.0), 5 mM; Mops (pH7.0), 100 mM; MgCl_2_, 100 mM; CaCl_2_, 700 μM; MnCl_2_, 2 mM; FeCl_3_, 50 μM; ZnCl_2_, 700 μM; thiamine, 2 μM; glycerol, 0.5%; glutamic acid, 0.5%; tryptophan, 50 μg/L; phenylalanine, 50 μg/L; 20 g agar in 1 L of distilled water; pH 7.0), GFP‐labeled R31 and the strain to be tested were inoculated in the center of the plate in triplicate in a ratio of 1:1, 1:2, or 2:1 (v/v). The plates were incubated for 3 days at 37°C, and the results of the experiment were recorded. 100 μL of suspension for GFP‐labeled R31 or the tested strain (diluted 100‐fold with sterile water) was spread onto an MSgg plate. Then, 6 μL of a third‐level culture suspension of GFP‐labeled R31 or the tested strain (OD_600_ = 0.5) was dropped onto the plate. After incubation at 37°C for 14–24 h, the size of the halo between the two strains was recorded. Determination of interactions between non‐*Bacillus* strains and R31 was performed as follows: R31 and tested strains were streaked into TSA plates for activation overnight. Single colonies were picked into 5 mL liquid TSB and incubated overnight at 37°C with shaking [16]. Sterile water was used to adjust the OD_600_ to 0.1. In triplicate, 1 μL of R31 and the strain to be tested were spotted on both sides of a square Petri dish seven times, with the inoculation sites getting closer to each other to form a V shape. After the Petri dishes were incubated at 30°C for 4 days, the experimental results were recorded by photography. Colony diameters were measured on a line orthogonal to the V‐shaped bisector.

### Biocontrol in potted banana plants

A single R31 colony was selected, placed into 100 mL NB medium, and cultured overnight at 37°C with shaking at 180 rpm. Then, 12 mL of the bacterial suspension was added to 200 mL of NB broth and cultured for 24 h at 180 rpm and 37°C. The number of cells in the broth was counted, and broths containing different individual strains were mixed with the R31 broth in equal proportions so that the titer of the mixed broth was 1 × 10^8^ cfu/mL. Then, 100 mL of the mixed broth was poured around the roots of each banana seedling, and a corresponding volume of distilled water was sprayed onto other plants as a control. After 10 days, R31 was applied once (200 mL of a 1 × 10^7^ cells/mL suspension) again. In 10 more days, the treated plants were inoculated with pathogen spores. The pathogenic FOC009 and FOC004 strains were removed from the refrigerator, and mycelia were cultured on a PDA medium for 1 week until the fungus covered the whole plate. A fungus plug was inoculated into oat culture medium and cultured for 1 week. The plate was eluted by 200 mL of double distilled water and filtered by four layers of gauze. The filtrate was collected and suspended. The pathogenic spore filtrate was diluted to a concentration of 1 × 10^5^ cells/mL, and 50 mL was applied to each banana seedling root. Forty days after inoculation, the disease status of the plants was investigated, and the disease index and biocontrol effect were calculated based on the wilting of the aboveground leaves and the Browning degree of the base section of the bulb and pseudostem. The disease index was calculated as ∑ (number of diseased plants at each level × representative value of each level)/(Total number of plants investigated × the highest representative value). The prevention and treatment effect (%) was calculated according to the formula (control disease index − treatment disease index)/control disease index × 100%.

### Statistical analyses

Statistical differences were performed in Microsoft Excel by Student's *t*‐test, R software 4.1.0 (R Core Team, R Foundation for Statistical Computing, Vienna, Austria. 2021. https://www.R-project.org), and SPSS (methods using least significant difference and Duncan (D)). Statistical significance was set at *p* < 0.05.

## AUTHOR CONTRIBUTIONS

**Ming‐Wei Shao**: Resources; project administration; writing—review and editing; writing—original draft; formal analysis. **Hao‐Jun Chen**: Writing—review and editing. **Ai‐Qin Huang**: Formal analysis. **Li Zheng**: Formal analysis. **Chun‐ji Li**: Formal analysis. **Di Qin**: Formal analysis. **Yun‐Hao Sun**: Formal analysis. **Zheng Lin**: Formal analysis. **Gang Fu**: Formal analysis; project administration. **Yan‐Hong Chen**: Formal analysis. **Yong‐Jian Li**: Formal analysis. **Zhang‐Yong Dong**: Formal analysis. **Ping Cheng**: Project administration; formal analysis. **Heru Pramono**: Formal analysis. **Guo‐Hui Yu**: Project administration; funding acquisition. **Zhi‐Min Xu**: Formal analysis; investigation. **Shuang Miao**: Writing—review and editing; writing—original draft; funding acquisition; project administration. **Kevin D. Hyde**: Formal analysis.

## CONFLICT OF INTEREST STATEMENT

The authors declare no conflicts of interest.

## 1 ETHICS STATEMENT

No animals or humans were involved in this study.

## Supporting information


**Figure S1:** Splicing and clustering of quality control of bacterial communities in control and R31‐treated groups.
**Figure S2:** Statistical plot of Tags and OTUs number of bacterial communities in control and R31‐treated groups.
**Figure S3:** Rarefaction curve of Alpha diversity.
**Figure S4:** Rank Abundance graph.
**Figure S5:** Venn diagram showing the overlap between core genera in the control and B31‐treated groups.
**Figure S6:** Heatmap of core microbiota distribution in two groups of soil samples.
**Figure S7:** Microbial interaction networks constructed for the core biocontrol genera and significantly interacting genera.
**Figure S8:** Heatmap illustrating the correlations between the core biocontrol genera and significantly interacting genera.
**Figure S9:** Prediction of differential bacterial communities function in control and R31‐treated groups based on PICRUSt2.
**Figure S10:** Analysis of phenotypic contribution based on Bugbase. Maps of species abundance for each phenotypic association.
**Figure S11:** Boxplots of the abundance of phenotypes of bacterial communities in control and R31‐treated groups based on Bugbase.
**Figure S12:** Determination of protease‐producing activity by beneficial strains.
**Figure S13:** Determination of chitinase‐producing activity by beneficial strains.
**Figure S14:** Determination of cellulase‐producing activity by beneficial strains.
**Figure S15:** Determination of *β*‐1,3 glucanase activity by beneficial strains.
**Figure S16:** Antagonism of *B. subtilis* R31 with endophytic bacteria in the roots of banana plants treated with *B. subtilis* R31.
**Figure S17:** The antagonistic experiment of *B. subtilis* R31 and root exophytic bacteria of banana plants treated with *B. subtilis* R31.
**Figure S18:** The antagonistic experiment of *B. subtilis* R31 and root bacteria in rhizosphere soil of banana plants treated with *B. subtilis* R31.
**Figure S19:** The antagonistic experiment of *B. subtilis* R31 and endophytic bacteria in the roots of banana plants in naturally healthy plots.
**Figure S20:** The antagonistic experiment of *B. subtilis* R31 and exogenous bacteria in the roots of banana plants in naturally healthy plots.
**Figure S21:** The antagonistic experiment of *B. subtilis* R31 and bacteria in rhizosphere soil of banana plants in naturally healthy plots.

**Table S1:** Data preprocessing, statistics and quality control in the microbiome under 12 samples.
**Table S2:** Tags details table in the microbiome under 12 samples.
**Table S3** Statistical table of Tags and OTUs number in the microbiome under 12 samples.
**Table S4:** Statistical table of the number of species annotation Tags in the microbiome under 12 samples.
**Table S5:** Stacked plot of species distribution at the phylum level.
**Table S6:** Species classification heatmap for phylum level classification.
**Table S7:** Alpha diversity index table in the microbiome under 12 samples.
**Table S8:** Summary results table for differential Alpha diversity.
**Table S9:** Kruskal statistic was used to test the differences at the phylum level in the microbiome under 4 groupings (2019_CK_vs_2019_T_vs_2020_CK_vs_2020_T).
**Table S10:** Kruskal statistic was used to test the differences at the genus level in the microbiome under 4 groupings (2019_CK_vs_2019_T_vs_2020_CK_vs_2020_T).
**Table S11:** Significant interactions between the core biocontrol genera and other bacterial genera in the CK control group.
**Table S12:** Significant interactions between the core biocontrol genera and other bacterial genera in the B. subtilis R31 ‐treated group.
**Table S13:** LEfSe statistic was used to test the differences in the microbiome under 4 groupings (2019‐CK_vs_2019‐T_vs_2020‐CK_vs_2020‐T).
**Table S14:** Indicator statistic was used to test the differences in the microbiome under 4 groupings (2019‐CK_vs_2019‐T_vs_2020‐CK_vs_2020‐T).
**Table S15:** Indicator statistic was used to test the differences at the genus level in the microbiome under 4 groupings (2019‐CK_vs_2019‐T_vs_2020‐CK_vs_2020‐T).
**Table S16:** Venn diagram based on OTU in the microbiome under 4 groupings (2019‐CK_vs_2019‐T_vs_2020‐CK_vs_2020‐T).
**Table S17:** Venn diagram at the phylum level in the microbiome under 4 groupings (2019‐CK_vs_2019‐T_vs_2020‐CK_vs_2020‐T).
**Table S18:** Venn diagram at the genus level in the microbiome under 4 groupings (2019‐CK_vs_2019‐T_vs_2020‐CK_vs_2020‐T).
**Table S19:** Kruskal statistic was used to test the differences at the pathway abundance information level in the microbiome under 4 groupings (2019_CK_vs_2019_T_vs_2020_CK_vs_2020_T).
**Table S20:** Kruskal statistic was used to test the differences at the pathway abundance information level in the microbiome under 4 groupings (2019‐CK_vs_2019‐T_vs_2020‐CK_vs_2020‐T).
**Table S21:** Tax4Fun functional annotation of pathway abundance information according to OTU species annotation and abundance information in the microbiome under 12 samples.
**Table S22:** Tax4Fun functional annotation of KO abundance information according to OTU species annotation and abundance information in the microbiome under 12 samples.
**Table S23:** BugBase analysis was used to analyze the phenotypic contribution of species at the phylum level in the microbiome under 4 groupings (2019‐CK_vs_2019‐T_vs_2020‐CK_vs_2020‐T).
**Table S24:** BugBase analysis was used to analyze differences (p‐values) between phenotype groups at the phylum level in the microbiome under 4 groupings (2019‐CK_vs_2019‐T_vs_2020‐CK_vs_2020‐T).
**Table S25:** The thresholds used in BugBase analysis of the phenotypic contribution of species at the phylum level in the microbiome under 4 groupings (2019‐CK_vs_2019‐T_vs_2020‐CK_vs_2020‐T).
**Table S26:** Phylogenetic affiliations of cultivatable bacteria isolated from the banana rhizosphere.
**Table S27:** The strains with the most biocontrol potential individual strain.
**Table S28:** The strains with the most biocontrol potential.
**Table S29:** Biocontrol effects of target strains on Fusarium wilt in potted banana plants.

## Data Availability

The data that support the findings of this study are openly available in BioSample accessions SAMN39308136, SAMN39308137, and SAMN3930813 at https://www.ncbi.nlm.nih.gov/bioproject/1062623, reference number PRJNA1062623. All the sequencing data have been deposited in NCBI under submission numbers SAMN39308136, SAMN39308137, SAMN39308138, SAMN39308139, SAMN39308140, and SAMN39308141, BioProject accession number PRJNA1062623, https://www.ncbi.nlm.nih.gov/bioproject/1062623. The data and scripts used are saved in GitHub https://github.com/shaomingwei-zk/Rhizosphere-Functional-Strains.git. Supplementary materials (figures, tables, graphical abstract, slides, videos, Chinese translated version, and update materials) may be found in the online DOI or iMeta Science http://www.imeta.science/imetaomics/.
