# Supplementary material for: Modulation of rhizosphere microbiota by Bacillus subtilis R31 enhances long‐term suppression of banana Fusarium wilt
Source: IMetaOmics. 2025 Mar 18;2(2):e70006. doi: 10.1002/imo2.70006 (PMC12806485; doi:10.1002/imo2.70006)
Supplement: Supplementary file 1 — Figure S1: Splicing and clustering of quality control of bacterial communities in control and R31‐treated groups. Figure S2: Statistical plot of Tags and OTUs number of bacterial communities in control and R31‐treated groups. Figure S3: Rarefaction curve of Alpha diversity. Figure S4: Rank Abundance graph. Figure S5: Venn diagram showing the overlap between core genera in the control and B31‐treated groups. Figure S6: Heatmap of core microbiota distribution in two groups of soil samples. Figure S7: Microbial interaction networks constructed for the core biocontrol genera and significantly interacting genera. Figure S8: Heatmap illustrating the correlations between the core biocontrol genera and significantly interacting genera. Figure S9: Prediction of differential bacterial communities function in control and R31‐treated groups based on PICRUSt2. Figure S10: Analysis of phenotypic contribution based on Bugbase. Maps of species abundance for each phenotypic association. Figure S11: Boxplots of the abundance of phenotypes of bacterial communities in control and R31‐treated groups based on Bugbase. Figure S12: Determination of protease‐producing activity by beneficial strains. Figure S13: Determination of chitinase‐producing activity by beneficial strains. Figure S14: Determination of cellulase‐producing activity by beneficial strains. Figure S15: Determination of β‐1,3 glucanase activity by beneficial strains. Figure S16: Antagonism of B. subtilis R31 with endophytic bacteria in the roots of banana plants treated with B. subtilis R31. Figure S17: The antagonistic experiment of B. subtilis R31 and root exophytic bacteria of banana plants treated with B. subtilis R31. Figure S18: The antagonistic experiment of B. subtilis R31 and root bacteria in rhizosphere soil of banana plants treated with B. subtilis R31. Figure S19: The antagonistic experiment of B. subtilis R31 and endophytic bacteria in the roots of banana plants in naturally healthy plots. Figure S20: The antag [file IMO2-2-e70006-s002.pdf]

Supplementary Information to

Modulation of Rhizosphere Microbiota by *Bacillus subtilis* R31 Enhances Long-Term Suppression of Banana Fusarium Wilt

Running title: R31 Promotes Rhizosphere-Functional Strains that Colonize Bananas to Control Fusarium Wilt

Ming-Wei Shao[1], Hao-Jun Chen[1], Ai-Qin Huang[1], Li Zheng[1], Chun-ji Li[1], Di Qin[1], Yun-Hao Sun[1], Zheng Lin[1], Gang Fu[2], Yan-Hong Chen[5], Yong-Jian Li[5], Zhang-Yong Dong[1], Ping Cheng[1], Heru Pramono[6], Guo-Hui Yu[1*], Zhi-Min Xu[3*], Shuang Miao[1*], Kevin D. Hyde[4]

[1]College of Agriculture and Biology, Key Laboratory of Green Prevention and Control on Fruits and Vegetables in South China, Ministry of Agriculture and Rural Affairs, Zhongkai University of Agriculture and Engineering, Guangzhou 510225, China

[2]Guangxi Key Laboratory of Biology for Crop Diseases and Insect Pests / Key Laboratory of Green Prevention and Control on Fruits and Vegetables in South China Ministry of Agriculture and Rural Affairs, Nannin, 530000, China

[3]Key Laboratory for Agro-ecological Processes in Subtropical Regions, Institute of Subtropical Agriculture, Chinese Academy of Sciences, Changsha, 410125, China

[4]Center of Excellence for Fungal Research, Mae Fah Luang University, Chiang Rai, 57100, Thailand

[5]Zhuhai Modern Agriculture Development Center, Zhuhai, 519090, China

[6]Laboratory of Fisheries Microbiology, Department of Marine Science, Faculty of Fisheries and Marine, Universitas Airlangga, Surabaya, 60115, Indonesia

*Correspondence: miao2008shuang@163.com (Shuang Miao), zhiminxusoil@isa.ac.cn (Zhimin Xu), ygh76411@zhku.edu.cn (Guohui Yu)

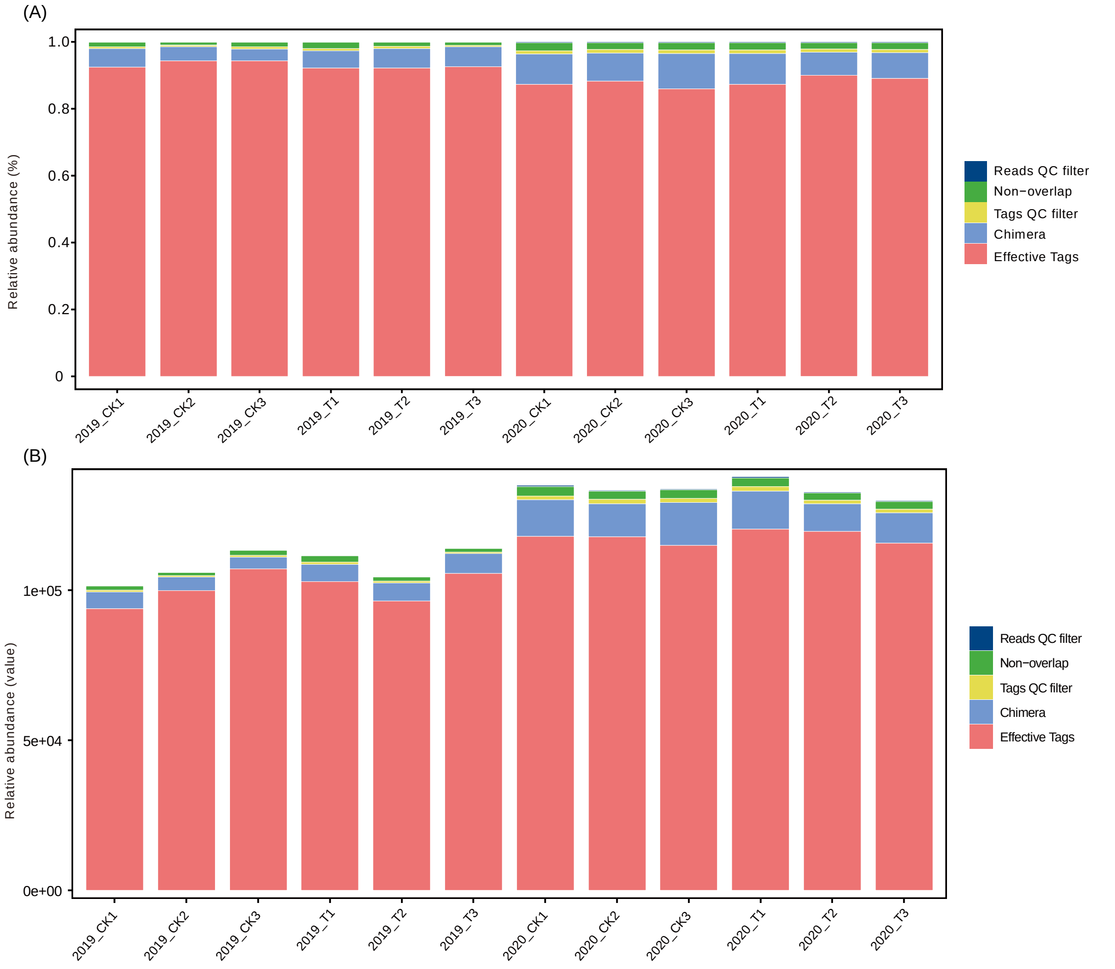

Figure S1 Splicing and clustering of quality control of bacterial communities in control and R31-treated groups. (A) Distribution of data preprocessing (percentage). (B) Distribution map of data preprocessing (numerical value). The abscissa of the stack plot is the sample classification name, the ordinate represents the percentage and value, and the different colors represent the data preprocessing classification. After raw reads were obtained by sequencing, we first filtered the low-quality reads, then assembled them, spliced the double-end reads into tags, and then filtered the tags. The obtained data was called clean tag. Clustering was performed based on clean tag to remove the chimera tag detected during the clustering process, and the obtained data was Effective tag. After OTU was obtained, OTU abundance statistics were performed based on Effective tag. Reads QC filter, low quality reads; Non-overlap, unassembled reads without overlap; Tag QC filter, tags that do not pass “tag filter”; Chimera, tag number of chimera; Effective tag, the number of tags of effective data.

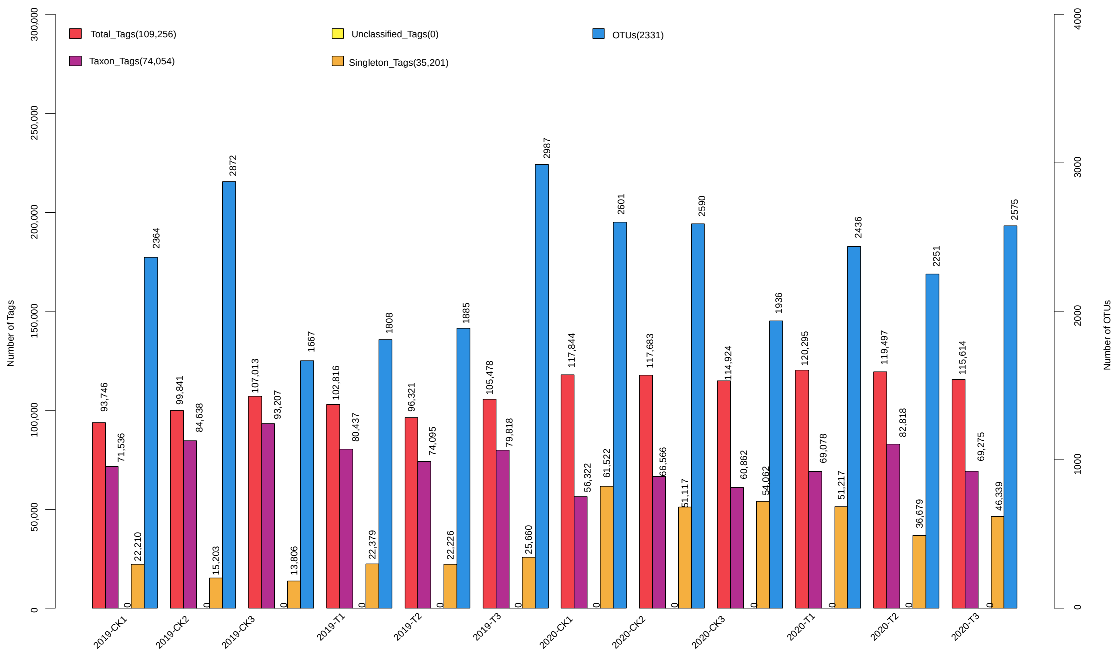

Figure S2 Statistical plot of Tags and OTUs number of bacterial communities in control and R31-treated groups. Based on the OTU abundance information and species annotation information, the overall characteristics of OTU, low-abundance OTU and Tags annotation of each sample were statistically summarized, and the statistical results were made into graphs. The abscissa of the plot is the sample classification name, the ordinate represents the value, and the different colors represent the data of OTU abundance.

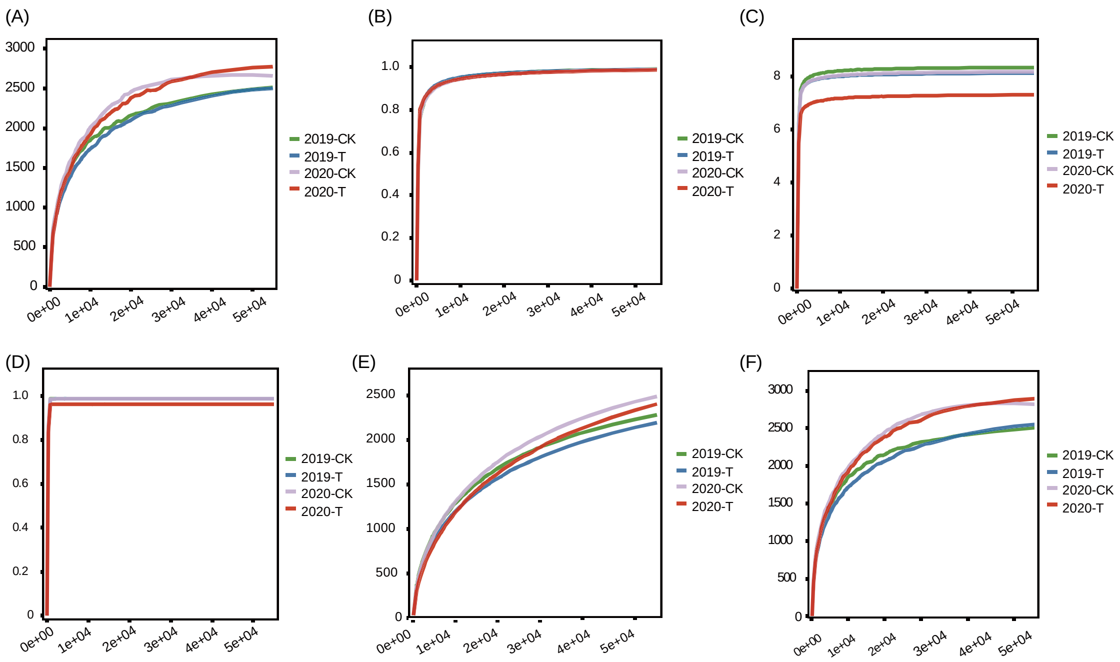

Figure S3 Rarefaction curve of Alpha diversity. Alpha diversity measured by the richness (A), Chao1, (B) Good coverage, (C) Shannon, (D) Simpson, (E) Sobs and (F) Ace indices. A certain amount of sequencing data is randomly extracted from samples, and their alpha diversity index values are counted. The horizontal axis represents the amount of sequencing data and the vertical axis represents the corresponding alpha diversity index. When the curve flattens or reaches a plateau, the sequencing depth can be considered to have basically covered all species in the sample.

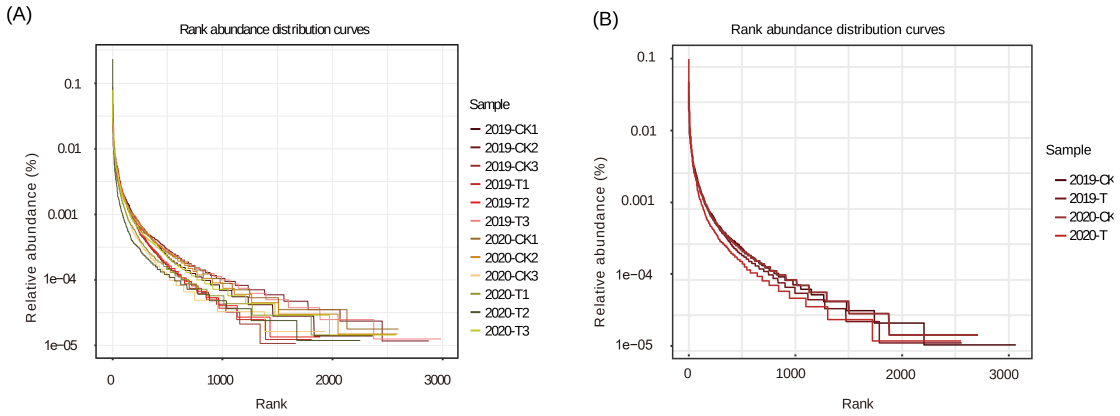

Figure S4 Rank Abundance graph. Rank Abundance distribution of sample (A) and grouping (B). The Rank Abundance curve can intuitively reflect the taxonomic richness and evenness contained in the sample. In the horizontal direction, the abundance of taxa was reflected by the width of the curve, the higher the richness of taxa, the larger the span of the curve on the horizontal axis. The smoothness of the curve in the vertical direction reflects the uniformity of the classification in the sample, and the flatter the curve, the more evenly distributed the species.

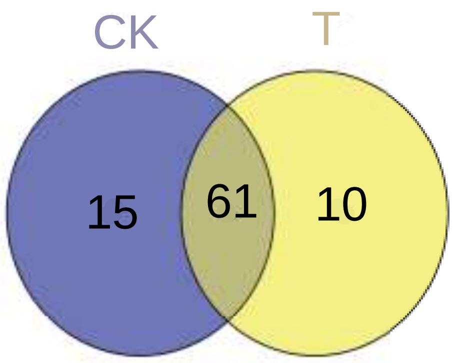

Genus	Core-in-CK	Core-in-T
Arthrobacter	Yes	Yes
Bacillus	Yes	Yes
Burkholderia	Yes	Yes
Enterobacter	Yes	Yes
Rhizobium	Yes	Yes
Sinomonas	Yes	Yes
Xanthomonas Campestris	No	No
Actinomycetes	No	No
Proteus	No	No
Propionibacterium	No	No
Bacteroides	No	No
Nesterenkonia	No	No

Figure S5 Venn diagram showing the overlap between core genera in the control and B31-treated groups. In Venn diagram, circles represent different groups, overlapping parts between circles represent the number of common species or genes, and non-overlapping parts represent the number of unique species or genes. A total of 76 core genus species were identified in CK group (naturally formed suppressive soil), and 71 core genus species were identified in T group (R31-induced antibacterial soil), of which 61 genera were common to the two groups of samples, while 15 core genus species were unique to CK group and 10 core genus species were unique to T group. Of the 12 genera tested experimentally, only 6 were core genera in both soil groups, and the other 7 were core genera that did not belong to either group (Table). 4 genera were not detected in the 16S (microbiome) amplicon data (Table in purple). Therefore, we focused on six common genera, which were the core genera shared by the two groups of soils. CK, suppressive soil; T, R31-treated.

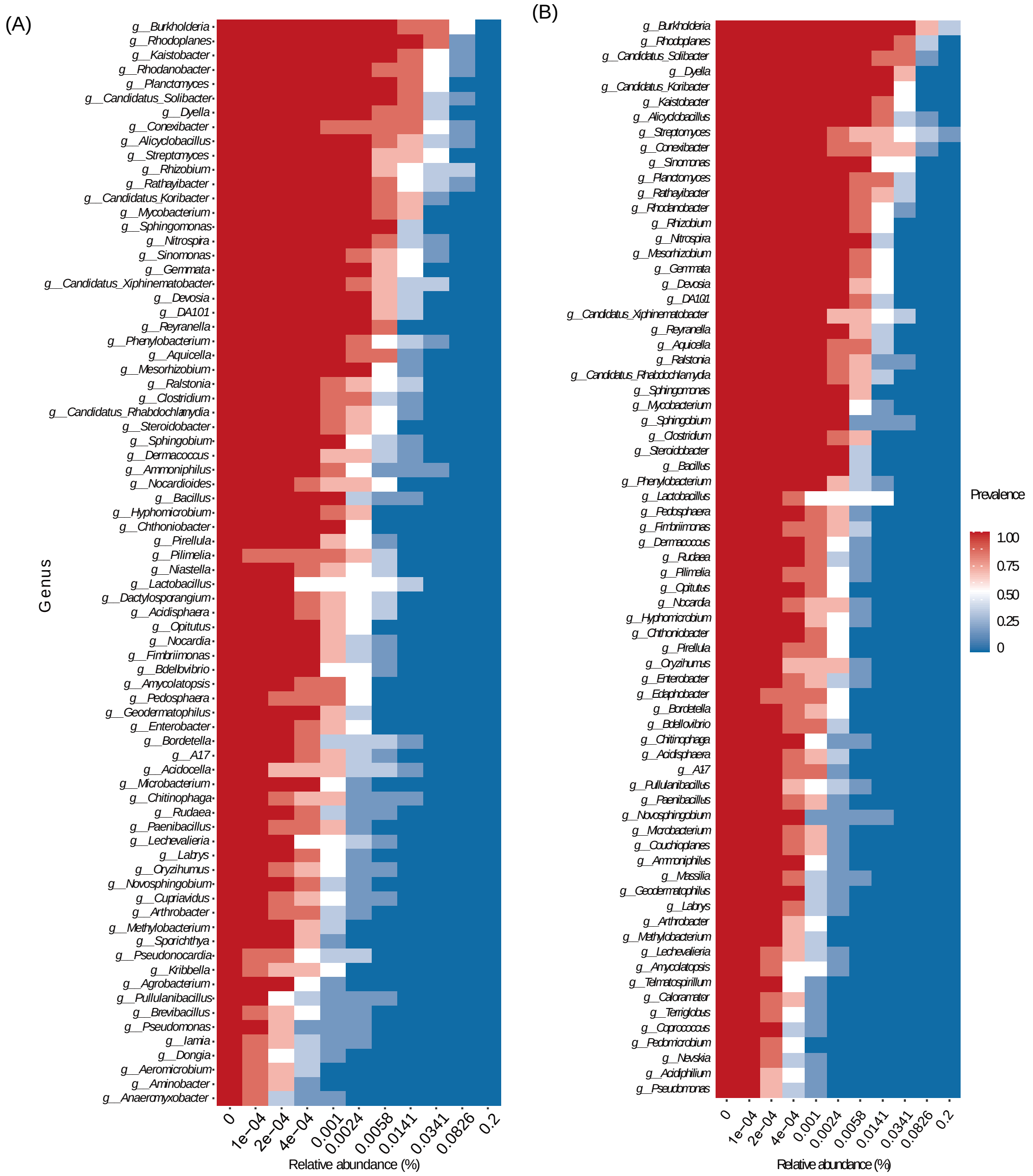

Figure S6 Heatmap of core microbiota distribution in two groups of soil samples. Heatmap illustrating the distribution of the core genera in all samples in control (A) and *B. subtilis* R31-treated (B) groups. The horizontal axis represents the threshold, which is the minimum proportion of genes for the phenotype, ranging from 0 to 0.2. The heatmap represents the corresponding calculated phenotypic abundance at different thresholds. When the threshold moves from left to right, the corresponding abundance shows a downward trend. The core genera detection parameter was set to “prevalence = 1.0” using the R package “phyloseq”, “RColorBrewer”, “ggplot2”, “microbiome”, “dplyr” based on the abundance matrix at the Genus level. The core degree of core species at different abundance thresholds was exported. CK, suppressive soil; T, R31-treated.

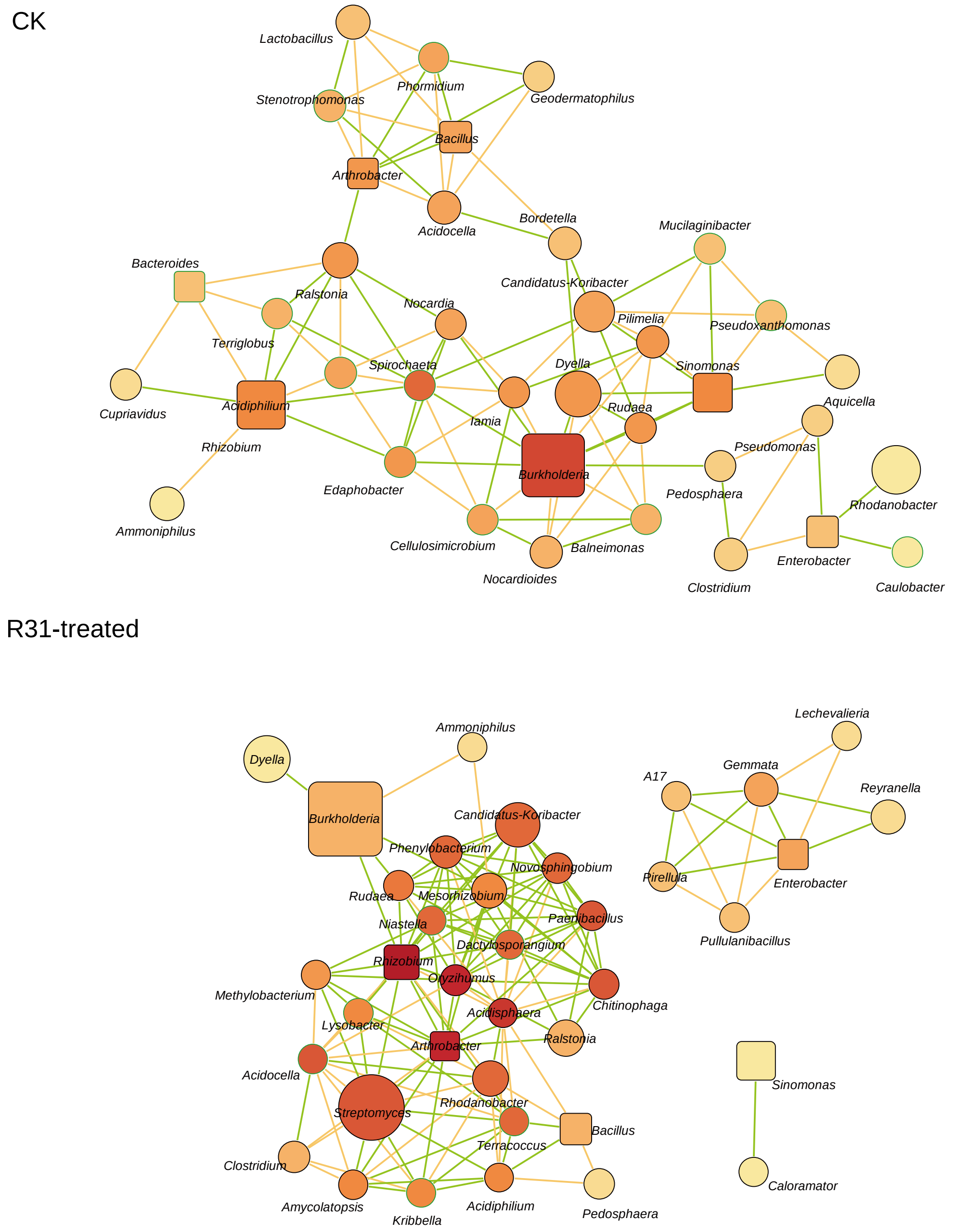

Figure S7 Microbial interaction networks constructed for the core biocontrol genera and significantly interacting genera. The nodal square indicates biocontrol bacteria, and the circle indicates other bacteria. Node black border represents core bacteria, green border represents non-core bacteria. Node size indicates high or low species abundance, and color from light to dark indicates low to high species centrality. The connection between the two nodes indicates positive correlation, orange indicates negative correlation, and the thickness of the line indicates the strength of correlation.

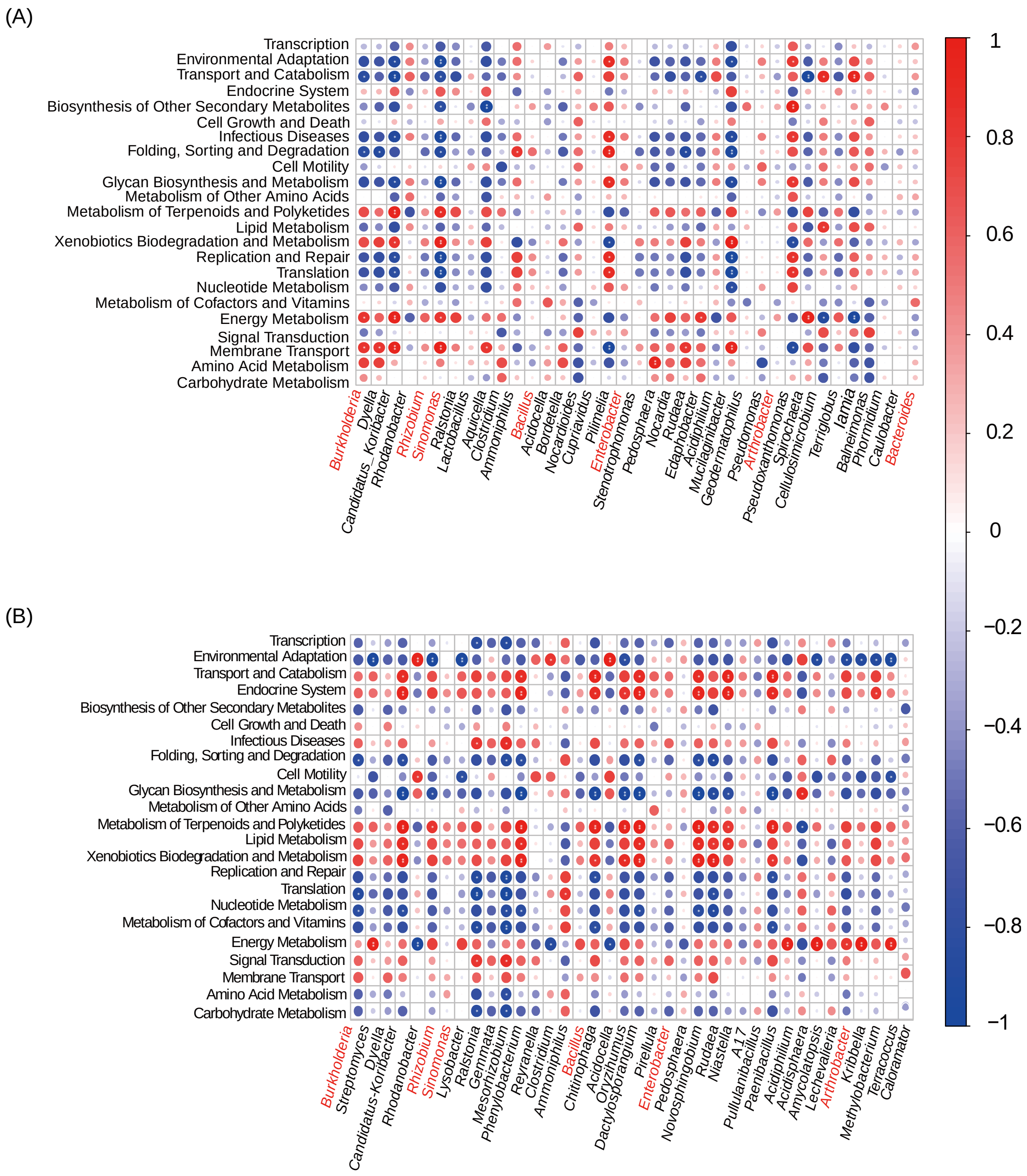

Figure S8 Heatmap illustrating the correlations between the core biocontrol genera and significantly interacting genera. Correlations in control samples (A) and R31-treated samples (B). * $p < 0.05$; ** $p < 0.01$ (Spearman correlation analysis). Genus names in red indicate core biocontrol genera.

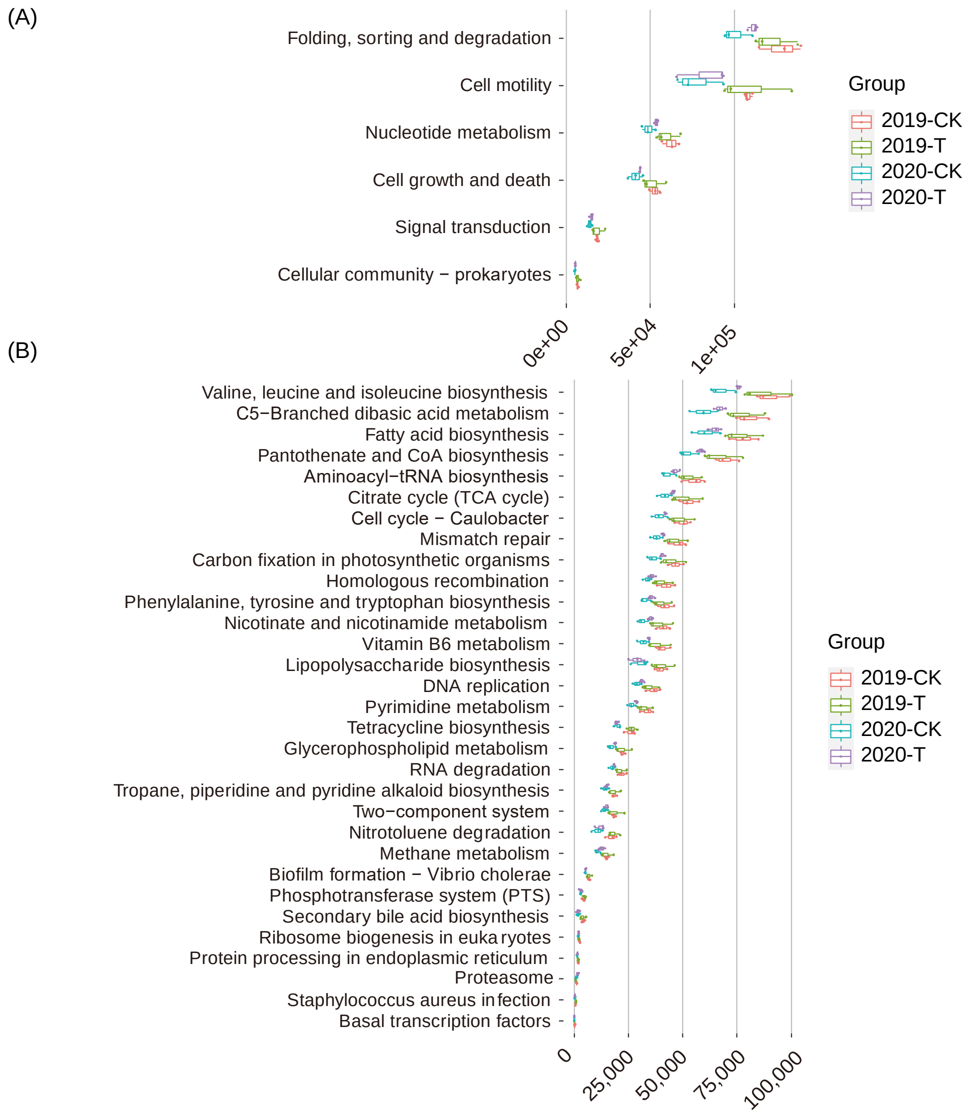

Figure S9 Prediction of differential bacterial communities function in control and R31-treated groups based on PICRUSt2. (A) Prediction of differential bacterial communities' function on level 2 of KEGG Pathway. (B) Prediction of differential bacterial communities' function on level 3 of KEGG Pathway. Based on OTUs species annotation and abundance information, functional annotation of KEGG Pathway of bacteria (16S) was performed using PICRUSt2 software. The abundance information of each Pathway was counted.

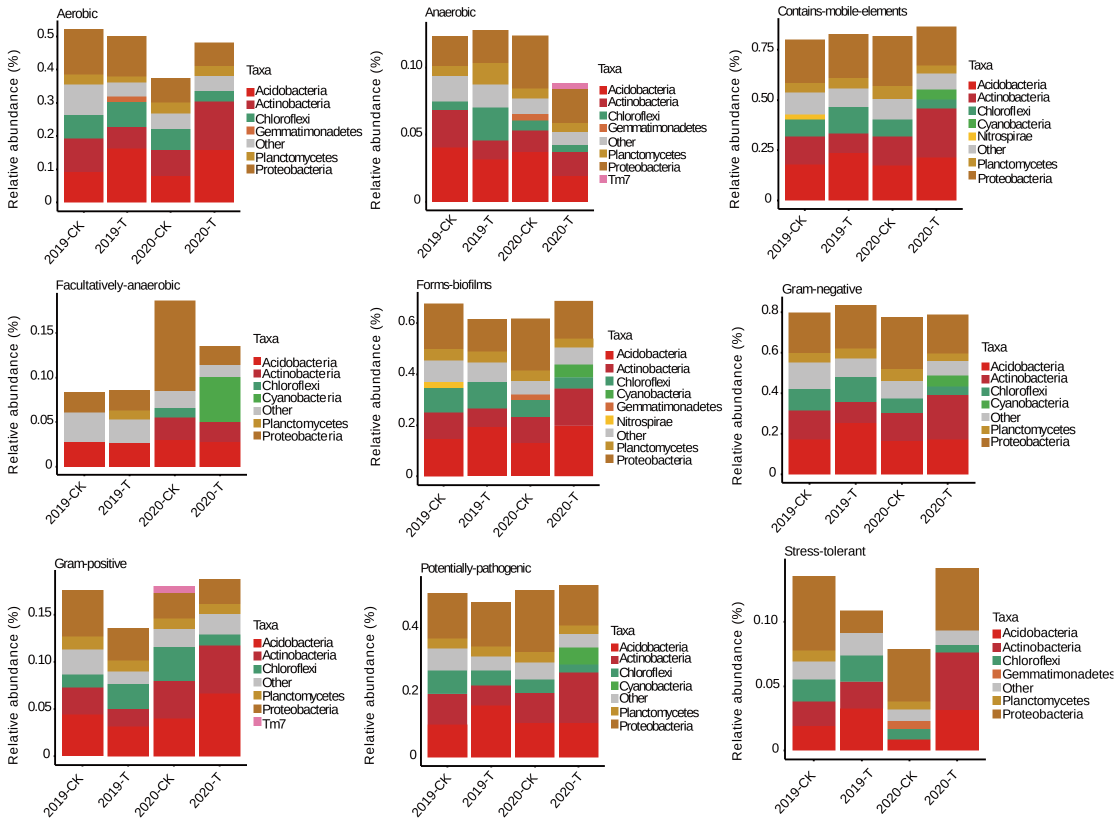

Figure S10 Analysis of phenotypic contribution based on Bugbase. Maps of species abundance for each phenotypic association. The abscyssus of the stacked plot shows the grouping of different samples, and the columns of different colors indicate the relative abundance of OTUs contributed by different species to the same phenotype. Based on the Greengenes database, we used Bugbase for phenotypic prediction of communities. By integrating the gene information of IMG, KEGG and PATRIC databases, they were divided into 7 main types: The bacteria are Gram Positive, Gram Negative, Biofilm Forming, Pathogenic, Mobile Element Containing), Oxygen Utilizing (including Aerobic type: Aerobic, Anaerobic type: Anaerobic, facultative anaerobic: Facultatively anaerobic) and Oxidative Stress Tolerant (Oxidative Stress Tolerant).

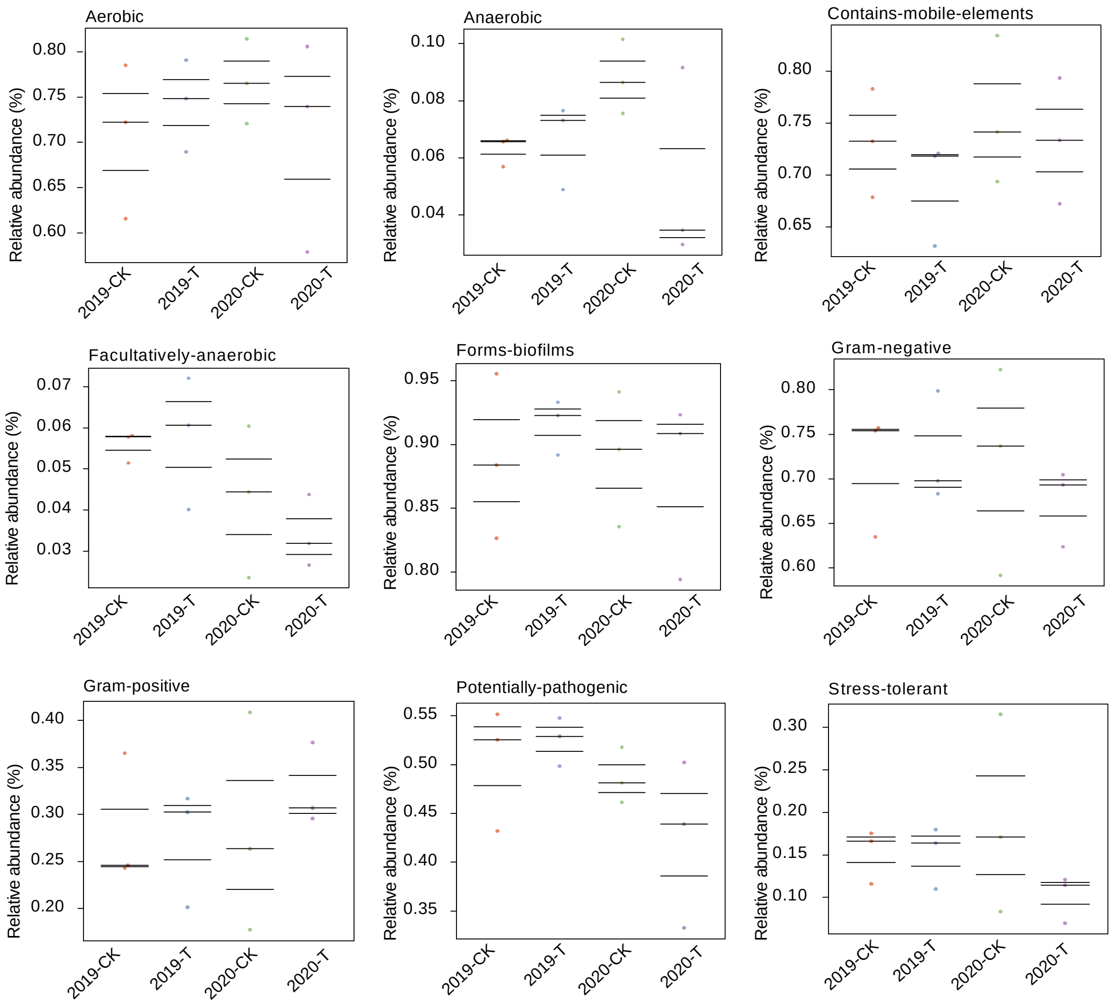

Figure S11 Boxplots of abundance of phenotypes of bacterial communities in control and R31-treated groups based on Bugbase. The horizontal axis of the boxplot is the grouping of samples, and the corresponding points are the abundance of samples in the group. The high, middle and low three lines represent the upper quartile, median and lower quartile of the abundance of samples in the group, respectively. By comparing the position of the box line in different groups, the abundance of different sample groups in different phenotypes can be compared. The phenotypic abundance results predicted by BugBase were used to perform rank sum test on pairwise grouping samples by R soft, and the difference between pairwise groups was shown by *p* value.

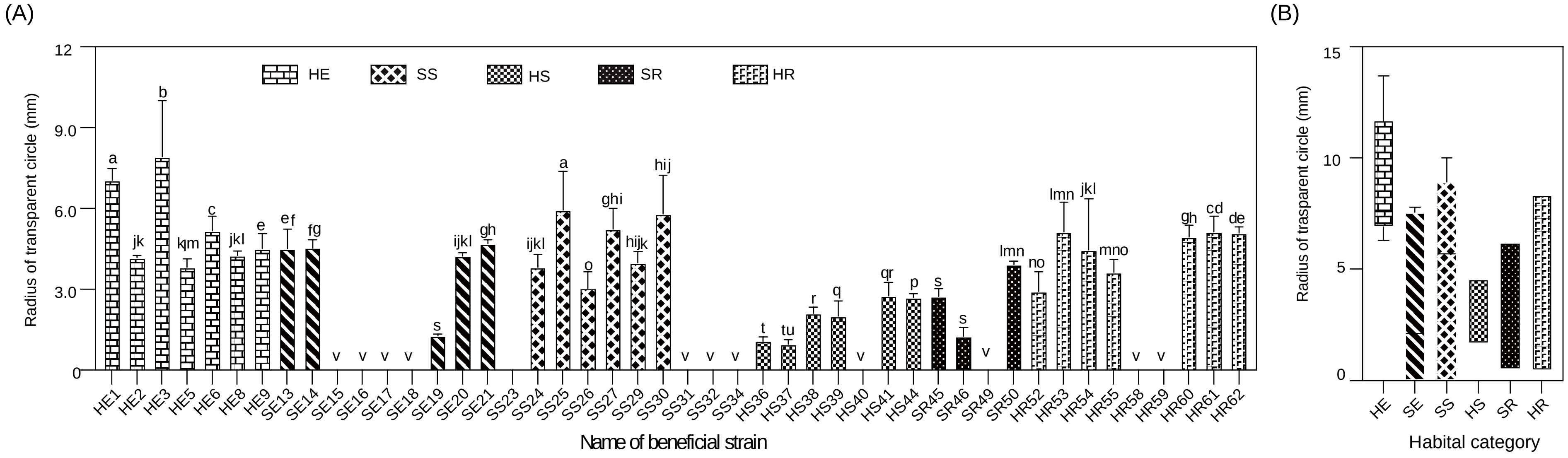

Figure S12 Determination of protease-producing activity by beneficial strains. (A) Radius of the transparent circles produced by protease activity of beneficial strains, used as an indicator of protease activity. (B) Radius of the transparent circles produced by protease activity of bacteria from different habitats. Different lowercase letters indicate significant differences between strains (p < 0.05). Data analysis was performed by SPSS (methods using Least Significant Difference (LSD) (L)) and Duncan (D)).

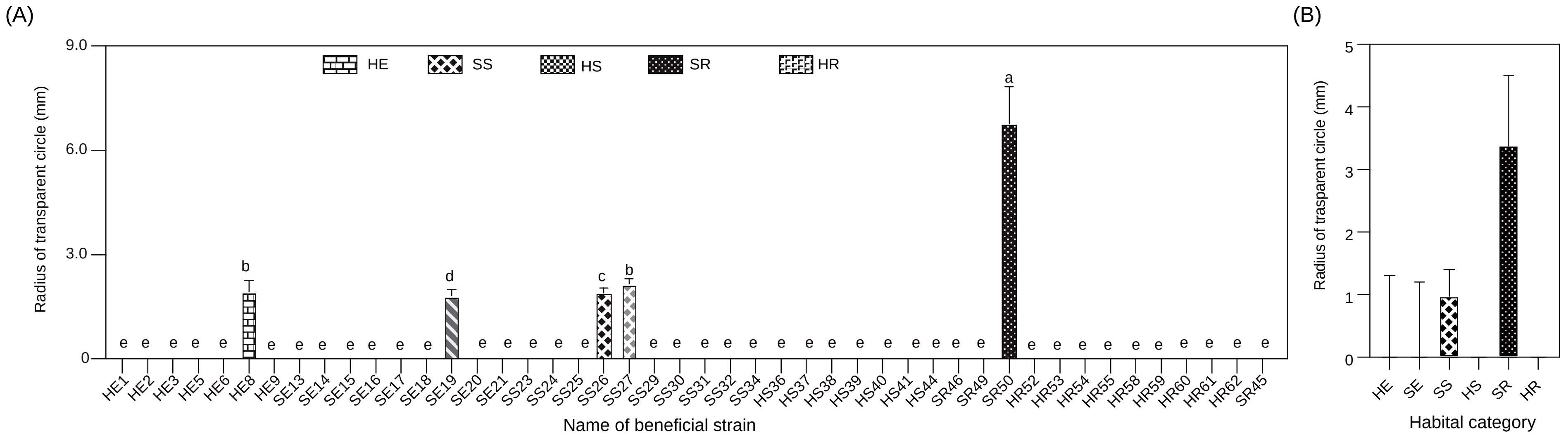

Figure S13 Determination of chitinase-producing activity by beneficial strains. (A) Radius of the transparent circles produced by chitinase activity of beneficial strains. (B) Radius of the transparent circles produced by chitinase activity of bacteria from different habitats. Different lowercase letters indicate significant differences between strains ($p < 0.05$). Data analysis was performed by SPSS (methods using LSD (L)) and Duncan (D)).

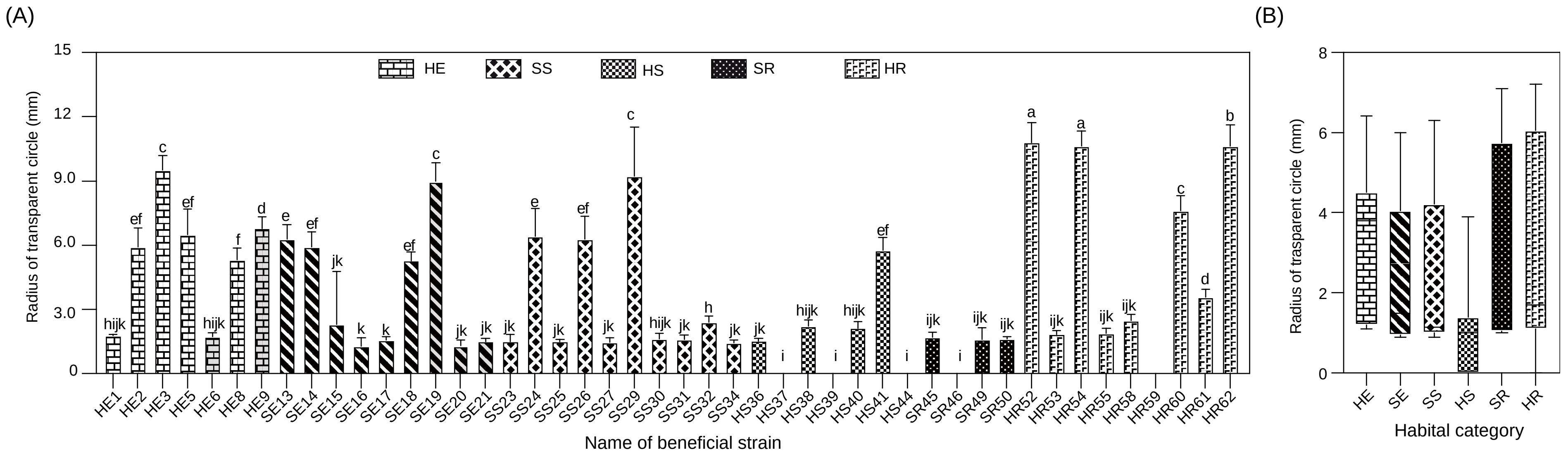

Figure S14 Determination of cellulase-producing activity by beneficial strains. (A) Radius of the transparent circles produced by cellulase activity of beneficial strains. (B) Radius of the transparent circles produced by protease activity of bacteria from different habitats. Different lowercase letters indicate significant differences between strains ($p < 0.05$). Data analysis was performed by SPSS (methods using LSD (L)) and Duncan (D)).

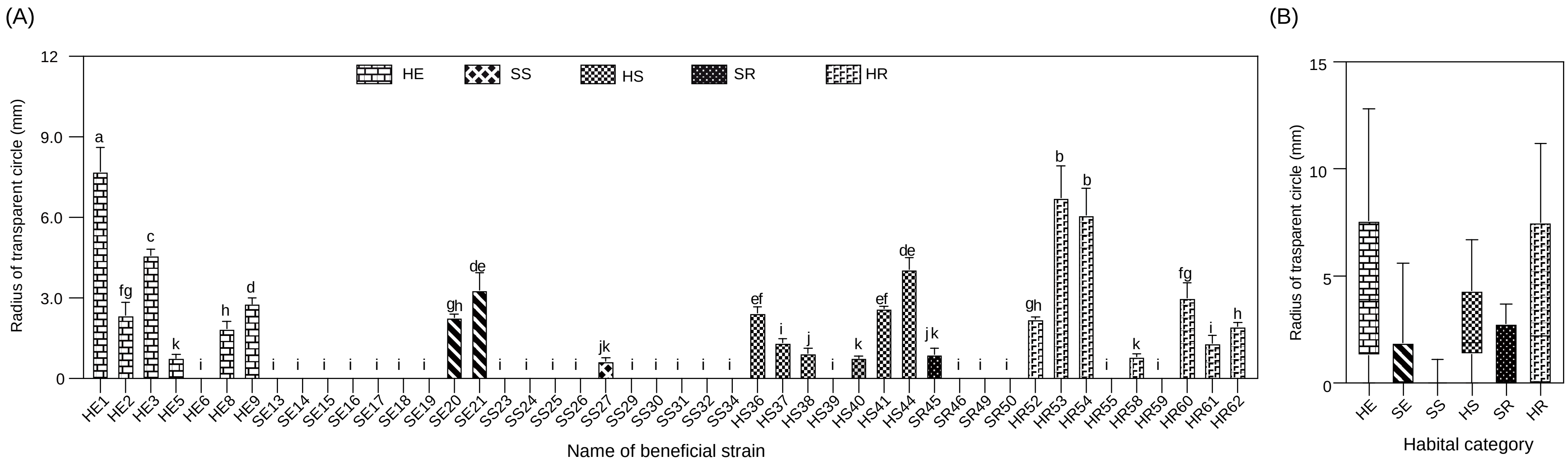

Figure S15 Determination of β-1,3 glucanase activity by beneficial strains. (A) Radius of the transparent circles produced by β-1,3 glucanase activity of beneficial strains; (B) Radius of the transparent circles produced by β-1,3 glucanase activity of bacteria from different habitats. Different lowercase letters indicate significant differences between strains ($p < 0.05$). Data analysis was performed by SPSS (methods using LSD (L)) and Duncan (D)).

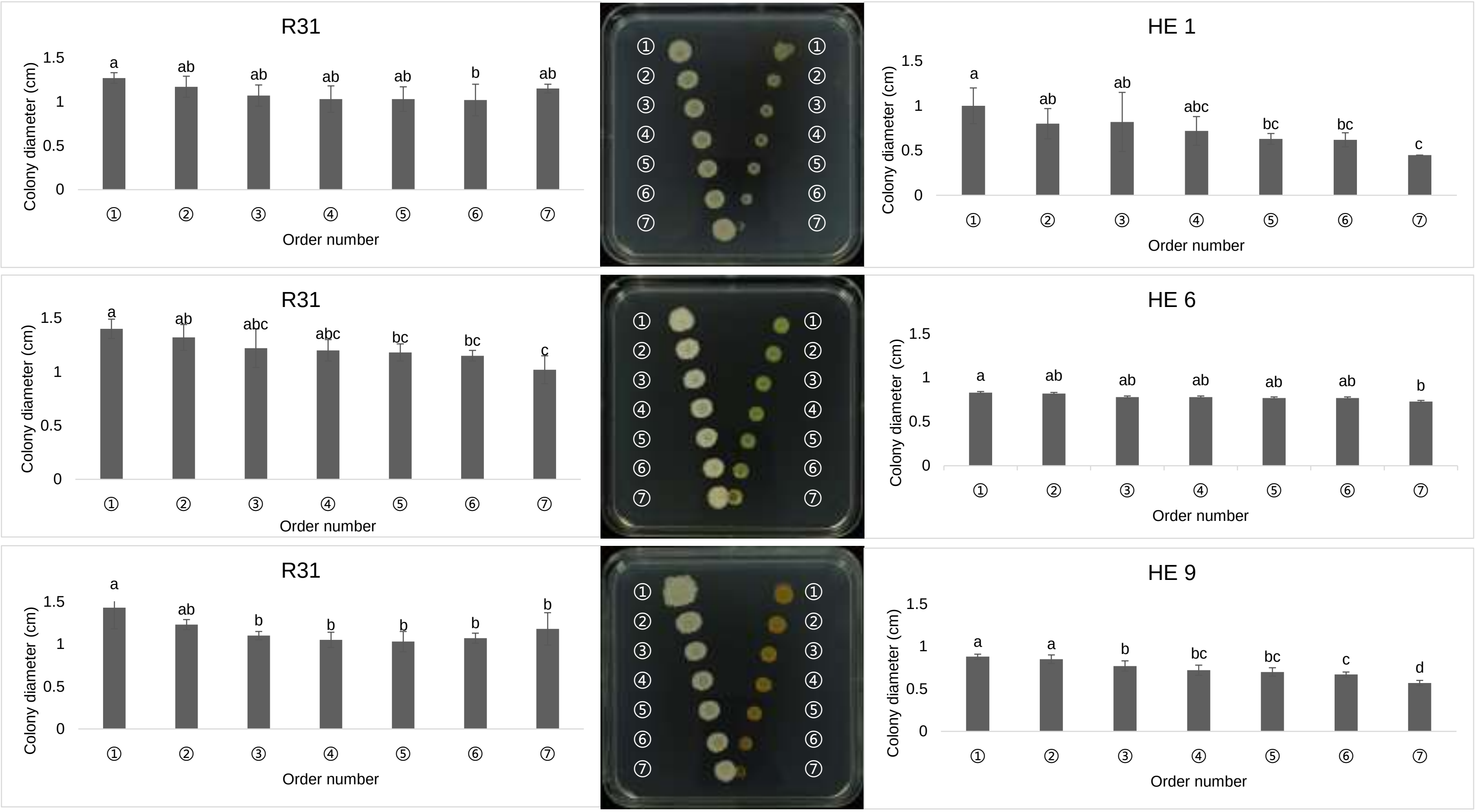

Figure S16 Antagonism of *B. subtilis* R31 with endophytic bacteria in the roots of banana plants treated with *B. subtilis* R31. Diameters of *B. subtilis* R31 colonies and the colonies of the indicated strains. Error bars represent the mean ± SD of the diameters. Different lowercase letters indicate significant differences (Duncan's new multiple range test, $p < 0.05$). The photographs in the middle show the antagonism between *B. subtilis* R31 and the indicated strains. Circled numbers represent the number of replicates performed.

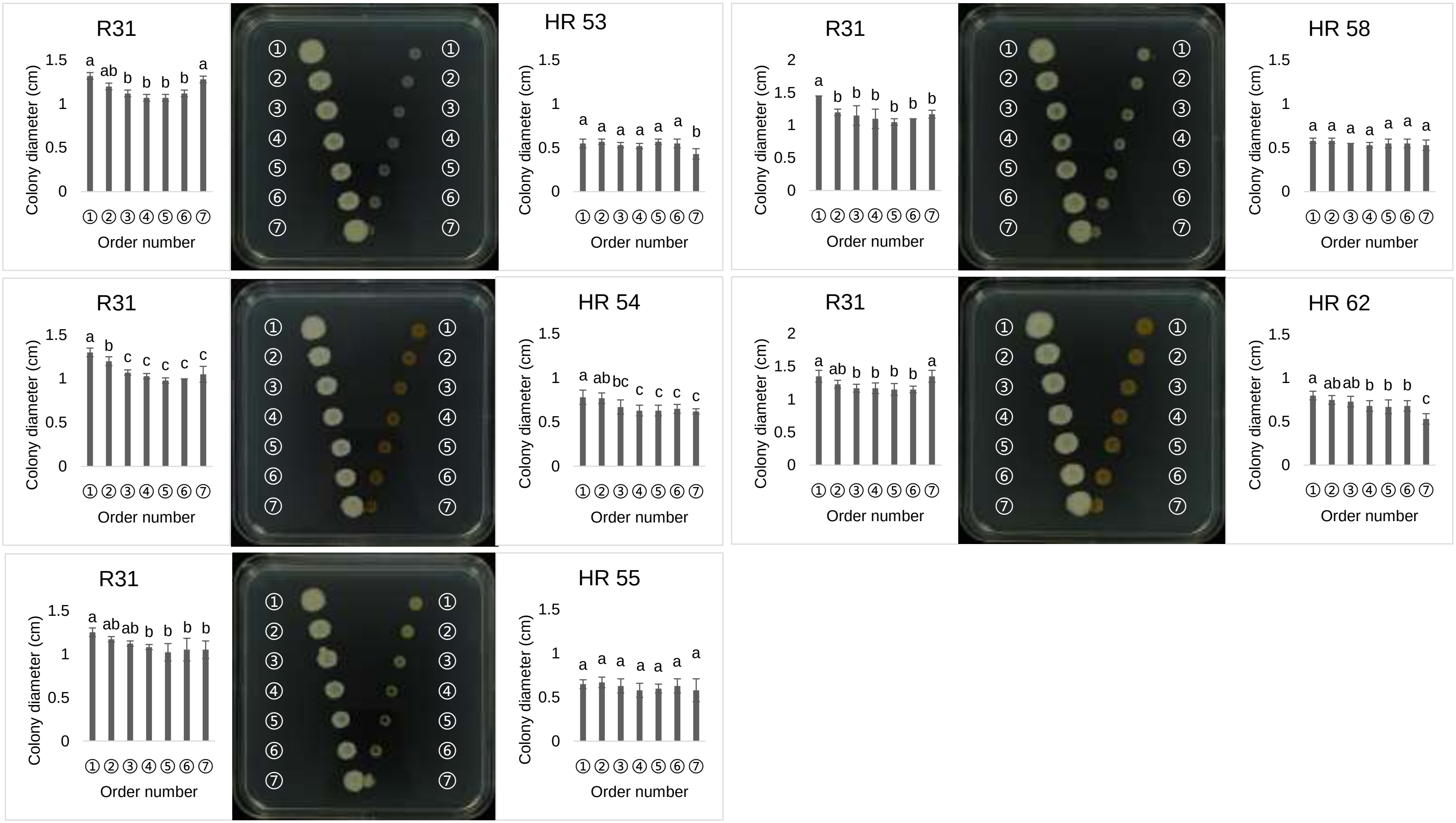

Figure S17 The antagonistic experiment of *B. subtilis* R31 and root exophytic bacteria of banana plants treated with *B. subtilis* R31. Diameters of *B. subtilis* R31 colonies and the colonies of the indicated strains. Error bars represent the mean ± SD of the diameters. Different lowercase letters indicate significant differences (Duncan's new multiple range test, $p < 0.05$). The photographs in the middle show the antagonism between *B. subtilis* R31 and the indicated strains. Circled numbers represent the number of replicates performed.

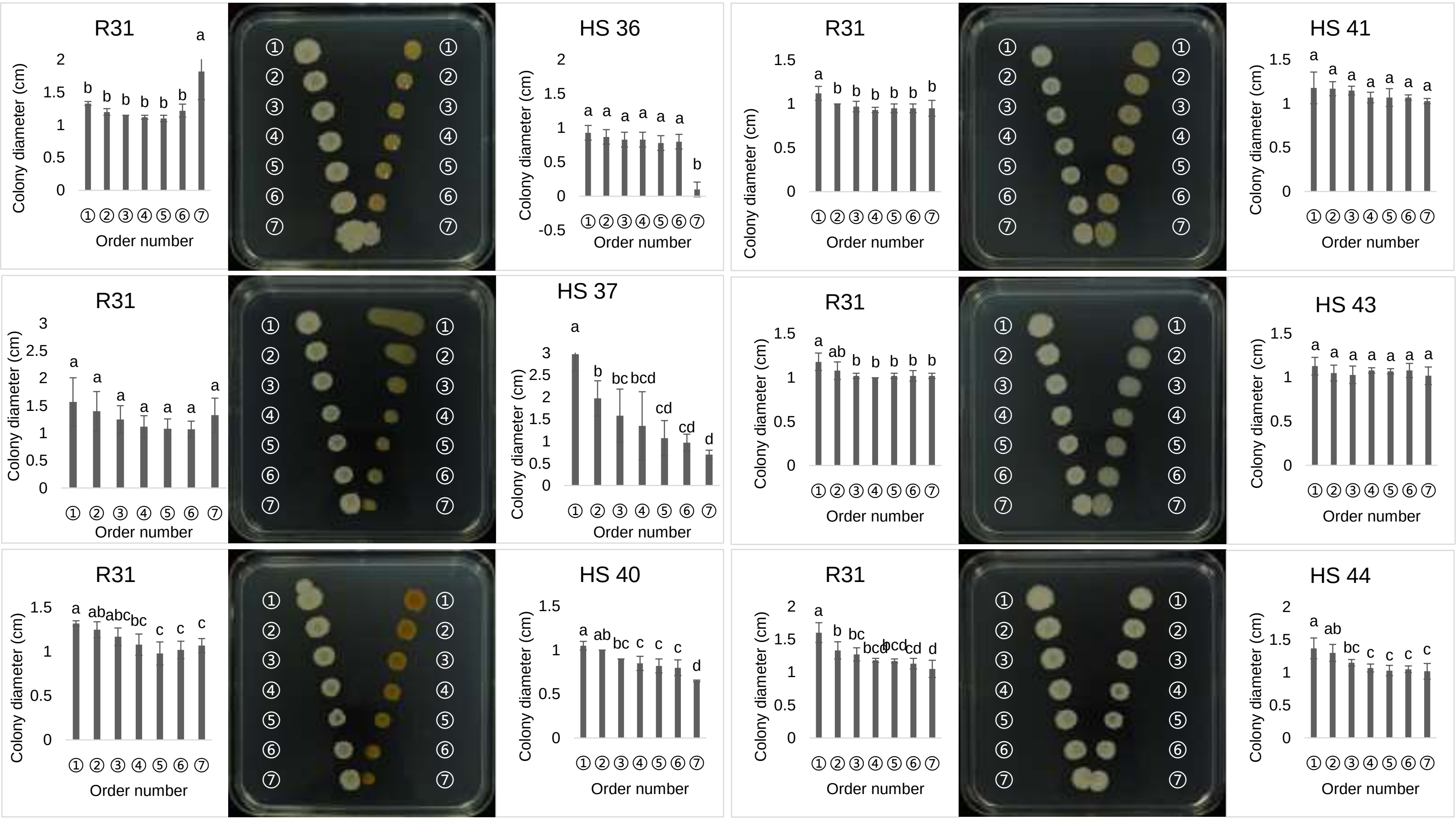

Figure S18 The antagonistic experiment of *B. subtilis* R31 and root bacteria in rhizosphere soil of banana plants treated with *B. subtilis* R31. Diameters of *B. subtilis* R31 colonies and the colonies of the indicated strains. Error bars represent the mean ± SD of the diameters. Different lowercase letters indicate significant differences (Duncan's new multiple range test, $p < 0.05$). The photographs in the middle show the antagonism between *B. subtilis* R31 and the indicated strains. Circled numbers represent tests varying the distance between R31 and the indicated strain.

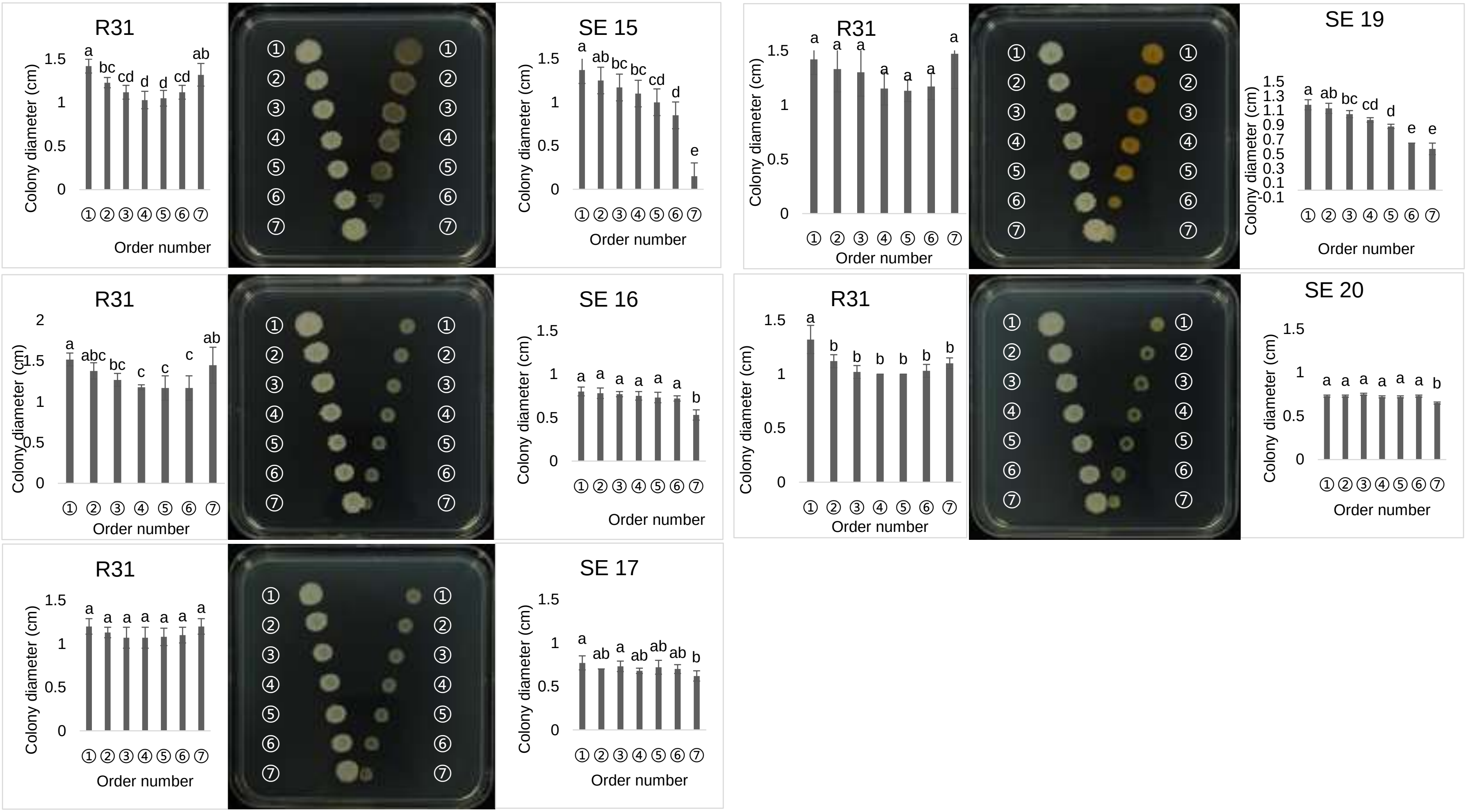

Figure S19 The antagonistic experiment of *B. subtilis* R31 and endophytic bacteria in the roots of banana plants in natural healthy plots. Diameters of *B. subtilis* R31 colonies and the colonies of the indicated strains. Error bars represent the mean ± SD of the diameters. Different lowercase letters indicate significant differences (Duncan's new multiple range test, $p < 0.05$). The photographs in the middle show the antagonism between *B. subtilis* R31 and the indicated strains. Circled numbers represent tests varying the distance between R31 and the indicated strain.

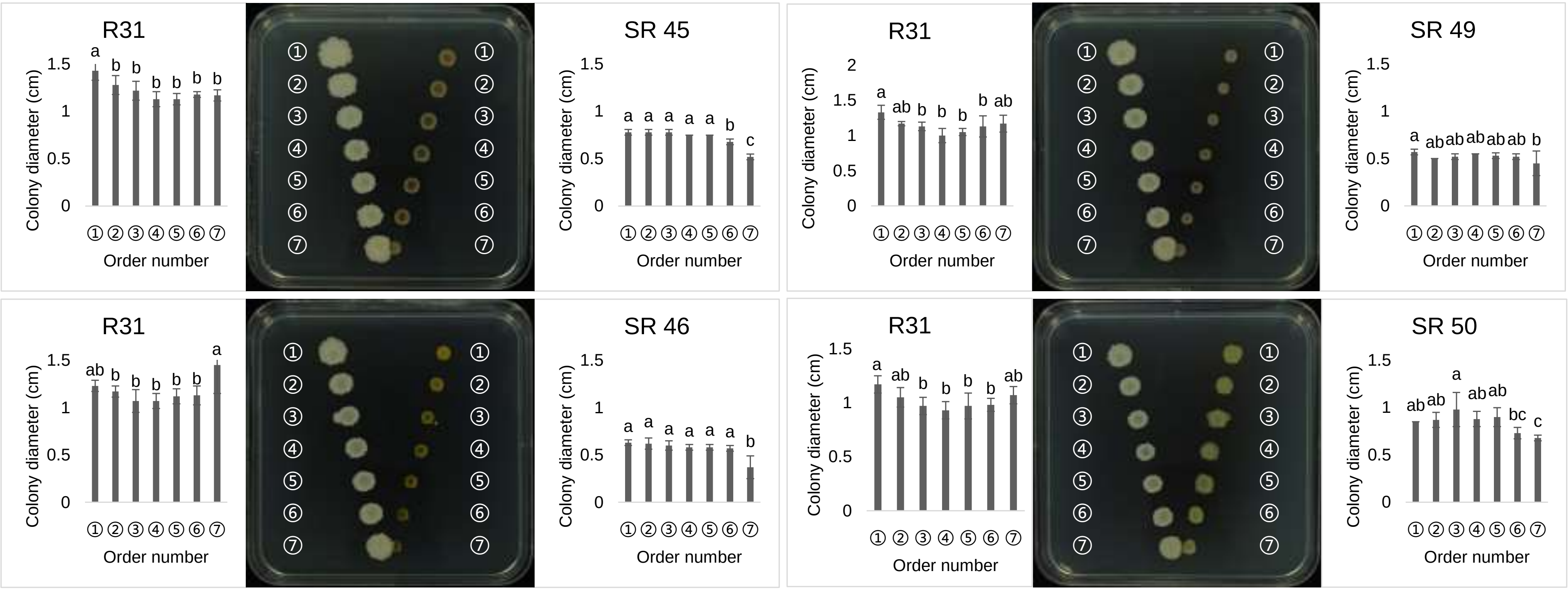

Figure S20 The antagonistic experiment of *B. subtilis* R31 and exogenous bacteria in the roots of banana plants in natural healthy plots. Diameters of *B. subtilis* R31 colonies and the colonies of the indicated strains. Error bars represent the mean ± SD of the diameters. Different lowercase letters indicate significant differences (Duncan's new multiple range test, $p < 0.05$). The photographs in the middle show the antagonism between *B. subtilis* R31 and the indicated strains. Circled numbers represent tests varying the distance between R31 and the indicated strain.

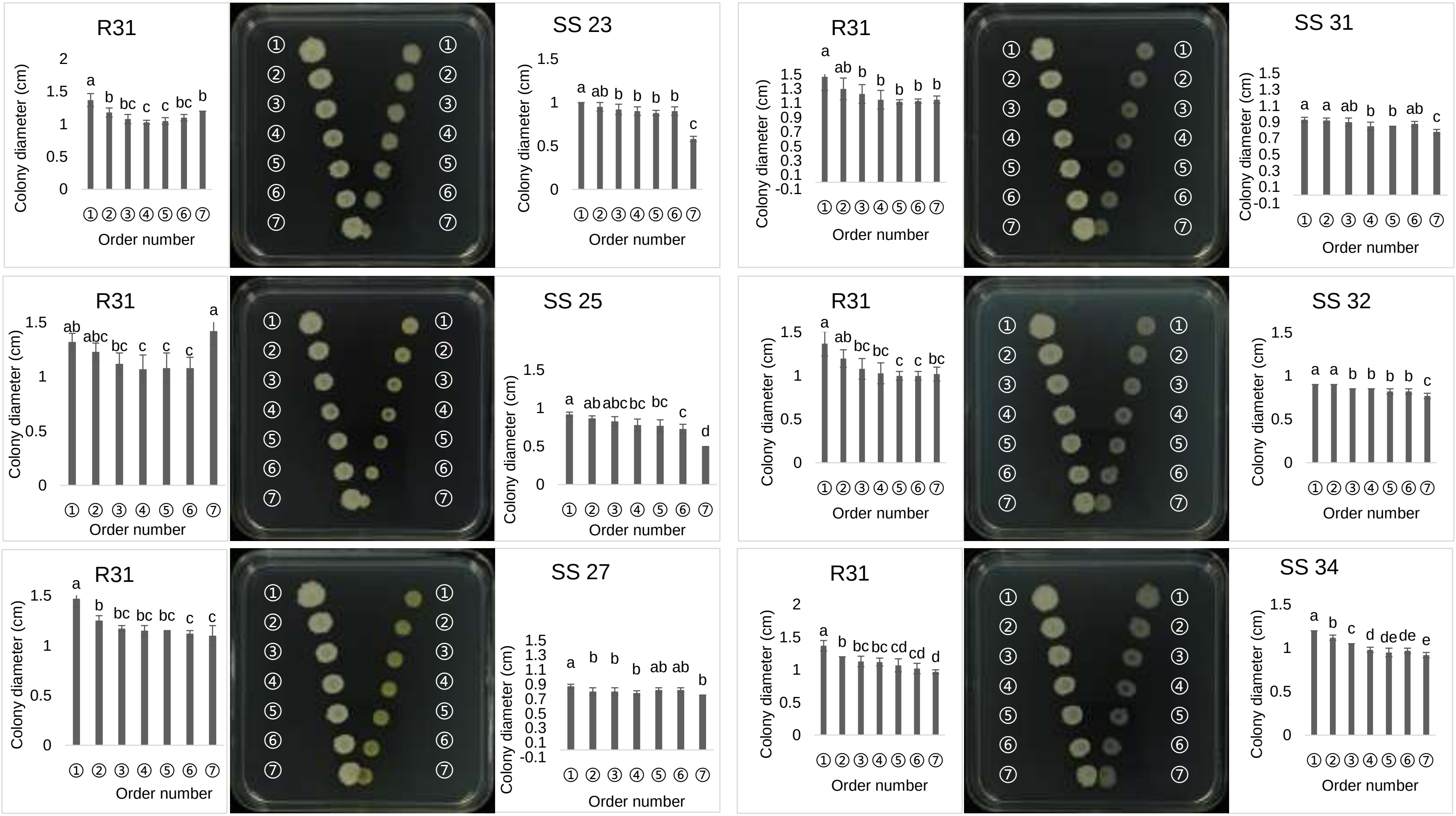

Figure S21 The antagonistic experiment of *B. subtilis* R31 and bacteria in rhizosphere soil of banana plants in natural healthy plots. Diameters of *B. subtilis* R31 colonies and the colonies of the indicated strains. Error bars represent the mean ± SD of the diameters. Different lowercase letters indicate significant differences (Duncan's new multiple range test, $p < 0.05$). The photographs in the middle show the antagonism between *B. subtilis* R31 and the indicated strains. Circled numbers represent tests varying the distance between R31 and the indicated strain.
